# Subnanometric Platinum–Germanium Clusters for Efficient Propane Dehydrogenation Catalysis

**DOI:** 10.1002/smll.73115

**Published:** 2026-03-17

**Authors:** Yuki Nakaya, Ken‐ichi Shimizu, Shinya Furukawa

**Affiliations:** ^1^ Institute for Catalysis Hokkaido University Sapporo Japan

**Keywords:** alloy clusters, hydrogen poisoning, nanoparticles, propane dehydrogenation, zeolite

## Abstract

Propane dehydrogenation has been a key technology with great industrial promise for meeting the growing global demand for propylene. Although much effort has been devoted to developing ideal catalysts that demonstrate high catalytic activity, selectivity, and durability at the same time, there have been few reports on the achievement of this goal due to a persistent tradeoff between activity and selectivity/stability. Herein, we report that subnanometric Pt─Ge alloy clusters encapsulated in pure silica MFI zeolite can break the activity–stability tradeoff in propane dehydrogenation. We also discovered that MnO*_x_* could act as an efficient co‐catalyst to reach the full potential of Pt─Ge alloy clusters by preventing hydrogen poisoning. The MnO*_x_*‐PtGe@MFI catalyst exhibited exceptionally high catalytic activity, selectivity, and durability in the absence of co‐fed hydrogen (for stabilization) at 600°C, exceeding those of other reported catalysts. Mechanistic study revealed that the combination of subnano‐downsizing, alloying Pt clusters with Ge, and hydrogen release by MnO*_x_* was the origin of the exceptional performance.

## Graphical abstract

Pt─Ge alloy clusters are regioselectively encapsulated within the sinusoidal channels of pure silica MFI zeolite (PtGe@MFI). The developed Pt─Ge alloy clusters exhibit exceptional performance with 36‐fold higher activity than the most stable catalyst ever reported and twice its stability, and with comparable activity to and 21‐fold higher stability than the most active catalyst (at 600°C).

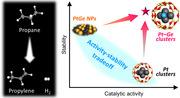

## 1 Introduction

Propylene is a key feedstock for the manufacture of various chemicals and has become extremely scarce because of the shale gas revolution. Global demand for propylene was approximately 110 million tons in 2022 and is forecast to reach more than 130 million tons in 2025 [1, 2, 3, 4], which illustrates its ever‐growing demand. Compared with other methanol‐to‐olefins and Fischer‐Tropsch‐to‐olefins reactions, propane dehydrogenation (PDH) produces propylene with high selectivity. Therefore, PDH is prevalent and a key technology that shows great industrial promise to meet the growing global demand for propylene. However, owing to its high endothermicity, PDH requires high operational temperatures (>600°C) to achieve a high propylene yield. Therefore, even Pt, which is among the most widely used metals in PDH catalysis owing to its high capability in C─H scissions (*e*.*g*., propylene production) and comparatively low capability in C─C cleavages (side reactions) [5, 6, 7], inevitably encounters severe problems during catalysis, such as low propylene selectivity, coke accumulation, and sintering. A highly sought goal in PDH catalysis is to fabricate highly active, selective, and durable catalysts. This is the key requirement from the standpoints of the efficient use of precious metals (*e*.*g*., Pt) and the elimination of the regeneration process, which would eventually reduce the overall cost of the PDH process. Although several materials show promise as state‐of‐the‐art Pt‐based catalysts, current materials generally suffer from intrinsic limitations and challenges [5, 6].

In general, researchers have focused intently on improving the selectivity and durability of Pt‐based catalysts, i.e., alloying Pt with less active or inert metals has typically been employed so that the dilution of large Pt─Pt ensembles effectively suppresses the undesired side reactions [5, 6, 7, 8, 9, 10, 11]. In our previous study, we reported the high catalytic performance of PtGe intermetallic and further improvement through multimetalization [8]. However, it is well known that the nanoparticulate Pt‐based alloys are indeed restricted by the scaling relationship between activity and selectivity/stability [5, 6, 11]. Therefore, even state‐of‐the‐art nanoparticulate alloy catalysts are compromised and suboptimal from the perspective of the activity–selectivity correlation [5, 6, 8, 11] and they often demonstrate low catalytic activity.

One possible candidate for overcoming this activity–selectivity/stability dilemma in nanoparticles is the downsizing of nanoparticles to subnanometric clusters. It has long been believed that propylene formation from propane (first and second C─H scissions) is generally unaffected by the geometric and electronic properties of Pt [5, 6, 7]. However, it has recently been reported that Pt clusters provide a much higher TOF in PDH owing to their unusual functionality [6, 12, 13], than do nanoparticulate or single‐atom Pt. In addition, the increase in the number of surface Pt sites that are accessible also contributes to increased productivity. However, the Pt cluster itself is detrimental to selectivity in PDH [14]. In this regard, an approach that combines alloying and downsizing into clusters to achieve high catalytic activity and selectivity is highly promising. Guided by this hypothesis, we focused on the synthesis of subnanometric Pt─Ge alloy clusters. However, it should be noted that subnanometric clusters have an inherent tendency to sinter at elevated temperatures, often irreversibly, which minimizes their surface energy [5, 15]. Therefore, it is typically necessary to adopt specific methodologies, i.e., spatial isolation (zeolite) [16, 17, 18], strong metal‐support interaction [19], or host–guest strategy [15], to retain the stability of the subnanometric clusters. In addition, alloying with Ge typically causes hydrogen poisoning that leads to lower catalytic activity [20]. Therefore, loading some co‐catalyst capable of hydrogen spillover, such as MnO*_x_* [21, 22], NbO*_x_* [23], or TiO*_x_* [24], adjacent to the Pt─Ge alloy cluster would be promising to avoid hydrogen poisoning.

Herein, we report for the first time a new class of heterogeneous catalysts, i.e., thermally stable subnanometric Pt─Ge alloy clusters that were regioselectively encapsulated within the sinusoidal channels of pure silica MFI zeolite (PtGe@MFI). The developed PtGe@MFI exhibited excellent activity, selectivity, and stability. In addition, we succeeded in further enhancing their catalytic performance by dispersing MnO*_x_* species in the vicinity of the Pt─Ge alloy clusters. As a result, the developed MnO*_x_*‐PtGe@MFI catalyst functioned as a highly active, selective, and stable PDH catalyst at high operational temperatures, which far exceeded existing catalysts. We believe that this work provides a novel dimension for the design of high‐performance catalysts for broader applications.

## 2 Results and Discussion

### 2.1 Identification of Subnanometric Clusters

**FIGURE 1:**
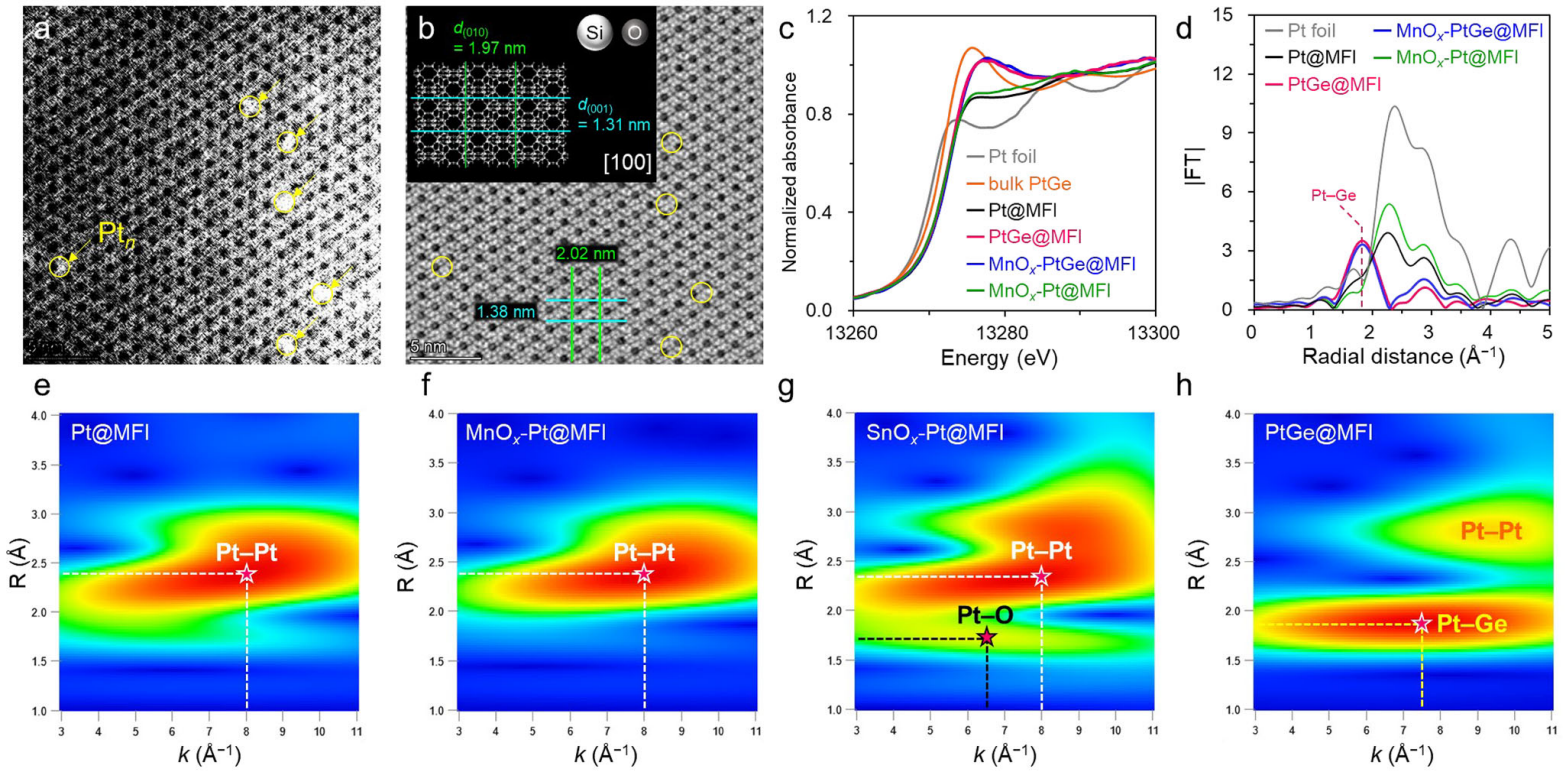
(a) modified HAADF‐STEM (drawn line mode), and the (b) corresponding iDPC images of MnO*_x_*‐PtGe@MFI (Mn/Ge/Pt = 3.3/1.8/1). Yellow circles correspond to the Pt clusters identified by STEM. Inset in (a) shows the structure of pure silica MFI viewed along [100] direction. Pt L_II_‐edge (c) XANES and (d) Fourier‐transformed EXAFS spectra of the Pt‐based samples. Pt L_II_‐edge wavelet transformed EXAFS signals of (e) Pt@MFI, (f) MnO*_x_*‐Pt@MFI, (g) SnO*_x_*‐Pt@MFI, and (h) PtGe@MFI.

PtGe@MFI and MnO*_x_*‐PtGe@MFI were synthesized using the one‐pot synthesis method for Pt@MFI in the literatures [16, 17, 18] with some modification (see Experimental details in Supporting Information for details). The X‐ray diffraction analysis confirmed the formation of the MFI structure (Figure S1). Inductively coupled plasma‐atomic emission spectroscopy (ICP‐AES) revealed that the mass loadings of Pt, Ge, and Mn were 0.46, 0.29, and 0.40 wt.%, respectively (Table S1). Prior to the characterizations and catalytic PDH reactions, the as‐prepared catalysts (e.g., MnO*_x_*‐PtGe@MFI‐Air) were reduced by H_2_ at 700°C for 5 h. High‐angle annular dark field scanning transmission electron microscopy (HAADF‐STEM) images and the corresponding elemental maps obtained using energy‐dispersive X‐ray (EDX) analysis confirmed that the Pt, Ge, and Mn species were homogeneously dispersed in the pure silica MFI zeolite (Figure S2). According to the particle size distribution, the mean particle size was 1.0 ± 0.3 nm (Figure S3). It is worth noting that undetectable clusters were not included in the particle size distribution; thus the actual mean size would be smaller than estimated. In this study, HAADF‐STEM images and integrated differential phase contrast (iDPC) images were measured simultaneously. Because iDPC analysis [25, 26, 27, 28] allows for observation of the zeolite framework owing to its high sensitivity to light elements (in this case; Si and O), the combination of HAADF‐STEM images (which are sensitive to heavy elements, such as Pt) and iDPC images provides valid information about the detailed location of Pt within the zeolite. Figure 1a,b are the modified HAADF‐STEM, and corresponding iDPC images of MnO*_x_*‐PtGe@MFI, respectively. The HAADF‐STEM and iDPC images revealed the locations of the subnanometric Pt‐based clusters and the 10‐membered ring (10MR) framework of the MFI along the [100] direction, respectively. Importantly, these clusters were not observed in the 10MR straight channels; however, they were observed in the overlapping zeolite framework. A similar result was observed from a different direction (Figure S3). These results strongly indicate that the clusters were regioselectively encapsulated within the sinusoidal channels of pure silica MFI [16, 17, 18]. We also synthesized the Pt@MFI, PtGe@MFI, and MnO*_x_*‐Pt@MFI catalysts in a similar manner, and these catalysts served as controls (refer to Supporting Method and Figures S4 and S5 for details). These catalysts showed particle size distributions similar to that of MnO*_x_*‐PtGe@MFI. Furthermore, we synthesized a SnO*_x_*‐Pt@MFI catalyst, in which SnO*_x_* species were located adjacent to the Pt clusters, as a reference (refer to Supporting Method and Figure S6 for details) [16, 17, 18].

### 2.2 In Situ XAFS Analysis

We then performed in situ X‐ray absorption fine structure (XAFS) measurements to obtain additional local structural information. Figure 1c,d shows the Pt L_II_‐edge X‐ray absorption near edge structure (XANES) spectra and Fourier‐transformed extended EXAFS (FT‐EXAFS) spectra, respectively, of Pt foil, bulk PtGe, Pt@MFI, PtGe@MFI, MnO*_x_*‐PtGe@MFI, and MnO*_x_*‐Pt@MFI (see Figure S7 for the Pt L_III_‐edge XANES spectra). Although Pt@MFI and MnO*_x_*‐Pt@MFI retained a metallic state, their XANES spectral features were distinct from those of Pt foil (Figure 1c). The FT‐EXAFS showed a significant decrease in their intensities compared to Pt foil, suggesting very small particle sizes (Figure 1d). Figure 1e–h shows the wavelet‐transformed EXAFS (WT‐EXAFS) [29, 30] signals of the Pt‐based MFI catalysts (see Figures S8 for the WT‐EXAFS signals of PtO_2_, Pt foil, and bulk PtGe). Pt@MFI and MnO*_x_*‐Pt@MFI showed an intense peak at *R* ≈ 2.5 Å and *k* ≈ 8 Å^−1^, characteristic of Pt─Pt bonds, while no signal attributable to Pt─O or Pt─Mn bonds was observed. The EXAFS curve‐fitting revealed that the coordination numbers (*CN*s) of the Pt─Pt scattering in Pt@MFI and MnO*_x_*‐Pt@MFI were 6.9 (*R*_Pt─Pt_ = 2.73 Å) and 6.7 (*R*_Pt─Pt_ = 2.73 Å), respectively (Figure S9 and Table S2). Similar curve‐fitting results were also obtained for the Pt L_III_‐edge EXAFS spectra (Pt@MFI: *CN*_Pt─Pt_ = 6.4, *R*_Pt─Pt_ = 2.72 Å; MnO_x_‐Pt@MFI: *CN*_Pt─Pt_ = 7.1, *R*_Pt─Pt_ = 2.73 Å, and Table S2). We calculated the Pt size–*CN*_Pt─Pt_ relationship using Pt*_n_* clusters and nanoparticles, which were taken from the Quantum Cluster Database (*n* = 3–55) [31], icosahedral (*n* = 60), dodecahedral (*n* = 140, 300, and 700), and cuboctahedral (*n* = 940–3000) models [32], respectively (Figure S10). The obtained Pt size–*CN*_Pt─Pt_ plot was well consistent with the size–*CN* correlation reported by Koningsberger et al. [33] The *CN*s_Pt─Pt_ of around seven in Pt@MFI and MnO*_x_*‐Pt@MFI corresponds to an average size of approximately 1 nm. Noted that the EXAFS curve‐fitting with a Pt─O or Pt─Mn scattering was unsuccessful. As detailed later in Mn K‐edge XAFS analysis, Mn species were present as oxides rather than in a metallic state. Thus, the combination of HAADF‐STEM and XAFS analyses strongly suggests that the majority of Pt species in both catalysts consisted of subnanometric Pt clusters and that there is no direct linkage between Pt clusters and MnO*_x_* species in MnO*_x_*‐Pt@MFI.

As a control catalyst that has direct linkage between Pt and oxide clusters, we also synthesized SnO*_x_*‐Pt@MFI [16, 17, 18] and measured its XAFS spectra. For the Pt L_II_‐edge XANES spectrum, the white line was shifted to a higher energy than those of Pt@MFI and MnO*_x_*‐Pt@MFI, which implies partial oxidation of Pt clusters (Figure S11). Unlike Pt@MFI and MnO*_x_*‐Pt@MFI, SnO*_x_*‐Pt@MFI showed a moderate peak at *R* ≈ 1.5 Å and *k* ≈ 6 Å^−1^ in the WT‐EXAFS, which corresponds to Pt─O scattering, in addition to an intense peak of Pt─Pt one (Figure 1g). This is likely attributable to the presence of SnO*_x_* species adjacent to the Pt clusters, as reported in previous studies [16, 17]. The presence of Pt─O scattering was also supported also by EXAFS curve‐fitting (Table S2). Thus, direct linkage between Pt and oxide clusters, if any, can be clearly detectable by EXAFS techniques. Therefore, the lack of such spectral features for MnO*_x_*‐Pt@MFI verifies that there is no direct linkage of Pt and MnO*_x_*.

Then, we analyzed PtGe@MFI and MnO*_x_*‐PtGe@MFI. In the Pt L_II_‐edge XANES spectrum of PtGe@MFI, the white line intensity was significantly increased by alloying Pt with Ge, a characteristic feature of Pt─Ge alloy formation [8], as seen in the bulk sample (Figure 1d) and previous reports on Pt─Ge NP systems [8, 34, 35]. In the Pt L_II_‐edge WT‐EXAFS of PtGe@MFI (Figure 1h), an intense signal appeared at *R* ≈ 1.9 Å and *k* ≈ 7.5 Å^−1^ in addition to Pt─Pt. Considering the higher *R* and *k* values than those observed for SnO*_x_*‐Pt@MFI, this can be assigned to backscattering by a larger and heavier element than oxygen, i.e., Pt─Ge. The presence of Pt─Ge and Pt─Pt bonds were further supported by EXAFS curve‐fitting: Pt─Ge and Pt─Pt scatterings at 2.40 Å (*CN*_Pt─Ge_ = 1.4) and 2.72 Å (*CN*_Pt─Pt_ = 5.4), respectively (Figure 1d; Table S2). These results strongly indicate the formation of a Pt‐rich Pt─Ge alloy. Herein, the summation of *CN*_Pt─Pt_ and *CN*_Pt─Ge_ was 6.8, which was close to the *CN*_Pt─Pt_ of 6.9 observed for Pt@MFI. Therefore, the Pt─Ge alloy clusters in PtGe@MFI have a size similar to that of the Pt clusters in Pt@MFI. Notably, MnO*_x_*‐PtGe@MFI showed XANES spectral features, an EXAFS oscillation, and a curve‐fitting result similar to those of PtGe@MFI (Figure 1c,d; Table S2). Combining our findings for MnO*_x_*‐Pt@MFI, it can be concluded that there is no direct chemical linkage between Pt─Ge clusters and MnO*_x_* species. Thus, MnO*_x_* in the vicinity of Pt─Ge alloy clusters did not change the electronic state or coordination environment of the Pt─Ge clusters.

The Ge K‐edge XANES and FT‐EXAFS spectra for MnO*_x_*‐PtGe@MFI are shown in Figures S12 and S13, respectively. After H_2_ reduction, the white line intensity decreased to some extent. Considering the formation of Pt─Ge alloy, it is likely that some portion of the Ge species was reduced to its metallic state to form the alloy, whereas excess Ge remained as an oxide, not participating in the alloying process. The XANES spectral curve of MnO*_x_*‐PtGe@MFI was reproduced through the linear combination fitting (LCF) of GeO_2_ (65.1%) and metallic Ge (34.9%) (Figure S14 and Table S3). The EXAFS curve‐fitting for MnO*_x_*‐PtGe@MFI showed Ge─O and Ge─Pt scatterings at 1.78 Å (*CN*_Ge─O_ = 3.5) and 2.48 Å (*CN*_Ge─Pt_ = 1.2), respectively (Figure S15 and Table S2). The Ge─O/Ge─Pt ratio was 2.9, which was similar to that estimated using LCF (Table S3). This also indicates the presence of Pt─Ge alloy clusters and GeO*_x_* species. We also conducted a XANES analysis of MnO*_x_*‐PtGe@MFI at the Mn K‐edge (see Figures S16 and S17 and Table S4 for details) in a similar fashion to that of the Ge K‐edge. MnO*_x_* species were partially reduced to lower valence states by the H_2_ reduction: MnO_2_ (16%), Mn_2_O_3_ (52%), Mn_3_O_4_ (17%), MnO (15%) → Mn_3_O_4_ (47%), and MnO (53%). The LCF with metallic Mn was unsuccessful, and this ruled out the presence of metallic or alloyed Mn. A temperature‐programmed reduction monitored with the use of Mn K‐edge XANES spectra and H_2_ consumption revealed that the partial reduction of Mn occurred at 400°C–600°C and ended by 700°C (Figures S18 and S19, respectively). The reduction behavior of Mn did not change in the presence of Ge (Figures S16 and S17), further supporting the conclusion that there is no direct linkage between Pt─Ge and MnO*_x_*. Thus, the proposed structure of MnO*_x_*‐PtGe@MFI is a composite of subnanometric Pt─Ge alloy and MnO*_x_* clusters in close proximity. It should be noted that this is the first report on the synthesis of the subnanometric Pt─Ge alloy cluster catalyst, which differs from the nanoparticulate PtGe alloys [8, 20, 35, 36, 37, 38] and Pt clusters linked to a Ge‐enriched zeolite framework (Pt─O─Ge bonding) [39].

### 2.3 Catalytic Performance and Mechanistic Study in PDH

**FIGURE 2:**
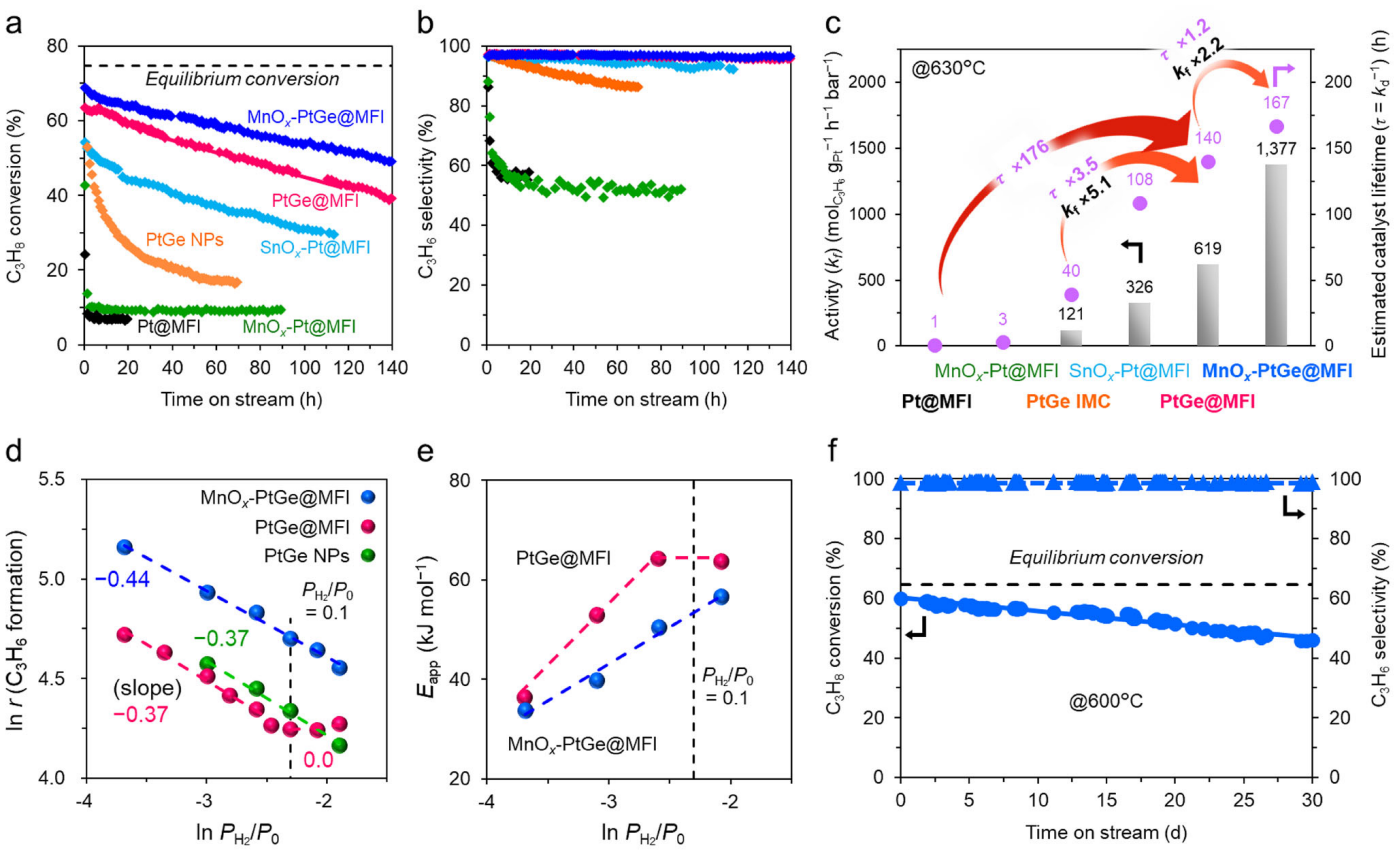
(a) C_3_H_8_ conversion and (b) C_3_H_6_ selectivity of Pt‐based catalysts in PDH at 630°C without co‐fed H_2_. Conditions: 30 mg of catalyst (150 mg for Pt@MFI and MnO*_x_*‐Pt@MFI), C_3_H_8_/He = 2.5/5.0, total flow rate *F* = 7.5 mL min^−1^. (c) Summary of catalytic activity (*k*_f_) and estimated catalyst lifetime (*τ* = *k*_d_^−1^) obtained during PDH at 630°C; *k*_f_ values were not correctly evaluated for Pr@MFI and MnO*_x_*‐Pt@MFI because of the extremely rapid deactivation at the beginning. Dependences of the (d) reaction rate and (e) *E*_a_ on *P*_H2_/*P*_0_. (f) Long‐term stability test of MnO*_x_*‐PtGe@MFI (Mn/Ge/Pt = 3.3/1.8/1) at 600°C without co‐fed H_2_. Conditions: 50 mg of catalyst, C_3_H_8_/He = 2.5/5.0, total flow rate *F* = 7.5 mL min^−1^.

Next, to evaluate the catalytic stability over a short time, PDH reactions were performed under an extremely harsh condition (630°C without co‐fed H_2_) (Figure 2a,b). For quantitative estimation of stability, we used the first‐order deactivation model [7]. Mean catalyst lifetime *τ* (reciprocal deactivation constant *k*_d_^−1^) serves as a key lifetime criterion, where a higher *τ* value represents a higher stability. We also employed the rate constant for the forward direction (*k*_f_) [8, 40, 41] to accurately estimate the catalytic activity even in near‐equilibrium regions. The corresponding equations for *k*_f_ and *τ* are described in the Experimental section. The *k*_f_ and *τ* values are summarized in Figure 2c. While Pt@MFI deactivated within 2 h (*τ* = 0.8 h) and plateaued lower than 10% conversion (see Text S1 for detailed explanation), PtGe@MFI exhibited much higher activity and stability (*k*_f_ = 619 mol_gPt_^−1^ h^−1^ bar^−1^, *τ* = 140 h). PtGe NPs (nanoparticulate intermetallic PtGe that was supported on silica, see Figure S20 for characterization), which was tested as a nanometric control, showed moderate activity and deactivated within a day (*k*_f_ = 121 mol_gPt_^−1^ h^−1^ bar^−1^, *τ* = 40 h). Regarding stability, alloying Pt clusters with Ge (Pt@MFI → PtGe@MFI) and downsizing PtGe NPs to clusters (PtGe NPs → PtGe@MFI) resulted in a 176‐fold and 3.5‐fold increase in the *τ* values, respectively (Figure 2c). Although we cannot completely rule out the influence of the confinement effect by zeolite, it is clearly very limited. This is evident because Pt@MFI was very unstable and the significant improvement was achieved by alloying with Ge (from Pt@MFI to PtGe@MFI). Thus, alloying Pt clusters with Ge is the primary key for the outstanding stability rather than the downsizing and/or the confinement effects [42].

Although PtGe@MFI exhibited high activity and stability, further enhancements can be achieved as shown below. It has been reported that alloying Pt NPs with Ge causes hydrogen poisoning to decrease catalytic activity [20]. The Pt and PtGe NPs showed that the reaction orders of the partial pressure of propane (*α*_C3H8_) were close to one. By contrast, that of H_2_ (*α*_H2_) changed from 0 to −0.5 upon Ge incorporation, suggesting that hydrogen atoms on Pt─Ge are strongly bound to the active Pt sites and compete with the dissociative adsorption of C_3_H_8_ (C─H scission, the rate‐determining step) [5, 6, 7] to lower the overall reaction rate [20]. Here, we performed a kinetic study under differential reaction conditions to discuss the influence of hydrogen on catalysis (the conversion was adjusted to levels much lower than the equilibrium). In our system, for PtGe@MFI and PtGe NPs, *α*_C3H8_ was close to one (Figure S21), which indicated that the C─H scission of C_3_H_8_ is the rate‐determining step, as is reported in the literature for PDH [5, 6, 7, 11]. We did not investigate *α*_C3H6_, since catalyst deactivation occurred even at 300°C by flowing propylene and hydrogen (Figure S22). Conversely, *α*_H2_ was negative (−0.37 for PtGe@MFI and PtGe NPs, Figure 2d), suggesting strong hydrogen poisoning to propane adsorption/activation as seen in a previously reported system [20]. Herein, PtGe@MFI showed an interesting trend when *P*_H2_/*P*_0_> 0.1: *α*_H2_ became zero, whereas PtGe NPs showed a negative *α*_H2_. These results indicate that hydrogen was saturated on PtGe@MFI probably because of the limited number of adsorption sites (Pt ensembles) due to the smaller size and Ge doping. Similar trends were also observed in the apparent activation energy (*E*_app_) of PDH at various *P*_H2_/*P*_0_ regions (Figure 2e). When *P*_H2_/*P*_0_ was below 0.1, a positive correlation was observed between *E*_app_ and *P*_H2_/*P*_0_, which is likely because the adsorbed hydrogen somehow modifies the electronic and/or geometric environment of the active Pt sites in Pt─Ge clusters to lower the activity. Therefore, to induce the potential ability of Pt─Ge alloy clusters for dehydrogenation, the coverage of hydrogen should be reduced by additional modification on the catalyst.

In this study, we focused on MnO*_x_* species, as they are known to be capable of hydrogen storage owing to reverse‐spillover [21], serving as a modifier to capture hydrogen. MnO*_x_*‐Pt@MFI showed catalyst stability comparable to that of Pt@MFI (*τ* = 2.9 h), whereas SnO*_x_*‐Pt@MFI exhibited higher stability, as reported in the previous studies [16, 17, 18]. Therefore, it is evident that direct modification of Pt clusters with oxides can somewhat enhance stability, whereas the indirect modification by MnO*_x_* does not. However, notably, MnO*_x_*‐PtGe@MFI exhibited the highest catalytic activity and stability among the synthesized catalysts (Figure 2a–c). The *k*_f_ and *τ* values were 2.2‐and 1.2‐fold higher than those of PtGe@MFI, respectively, indicating that an indirect effect by the neighboring MnO*_x_*, such as “hydrogen release”, enhanced the catalytic activity, but also stability. This highlights the validity and compatibility of subnano‐downsizing, alloying with Ge, and modification with MnO*_x_*.

Then, a kinetic study was performed to verify the role of MnO*_x_* on the behavior of hydrogen, revealing that *α*_H2_ was −0.44 over the entire *P*_H2_/*P*_0_ range for MnO*_x_*‐PtGe@MFI (Figure 2d). The absence of the zero‐order region that was observed for PtGe@MFI implies that hydrogen poisoning on the Pt─Ge clusters was mitigated. A similar trend was also observed in *E*_app_; the plateau region observed for PtGe@MFI (*P*_H2_/*P*_0_> 0.1), reflecting saturation coverage of hydrogen, did not appear for MnO*_x_*‐PtGe@MFI (Figure 2e). This is likely because the hydrogen coverage was lessened by hydrogen release to the “MnO*_x_* trap.” Moreover, the slope of the correlation was lower for MnO*_x_*‐PtGe@MFI than for PtGe@MFI, which supports the capability of MnO*_x_* against hydrogen poisoning. Additionally, we conducted H–D exchange by flowing a 1:1 mixture of H_2_ and D_2_ and estimated the apparent activation energies (*E*_app‐H2D2_) (Figure S23). When MnO*_x_* was present, the activation barriers were much lower (*e*.*g*., PtGe@MFI: 16.9 kJ mol^−1^ → MnO*_x_*‐PtGe@MFI: 8.3 kJ mol^−1^). These findings strongly elucidate that MnO*_x_* in the vicinity of Pt─Ge alloy clusters can hold spillover hydrogen from the clusters to lower the hydrogen coverage on Pt sites and explain why the catalytic activity was greatly enhanced. We also investigated the effects of Mn content and reduction time (see Figures S24–S27 and Text S2). While the stability did not depend largely on the Mn content, it was more enhanced when the reduction time was longer. Considering that the partial reduction of Mn ended before reaching 700°C (Figures S18 and S19), the longer reduction time needed for higher stability is attributed to migration of reduced MnO*_x_* species to an optimal distribution that maximizes the number of MnO*_x_* in the vicinity of metal clusters. Similar phenomenon was also reported for SnO_x_ in MFI [17]. This is why we performed the reduction pretreatment at 700°C for 5 h for catalytic tests.

**FIGURE 3:**
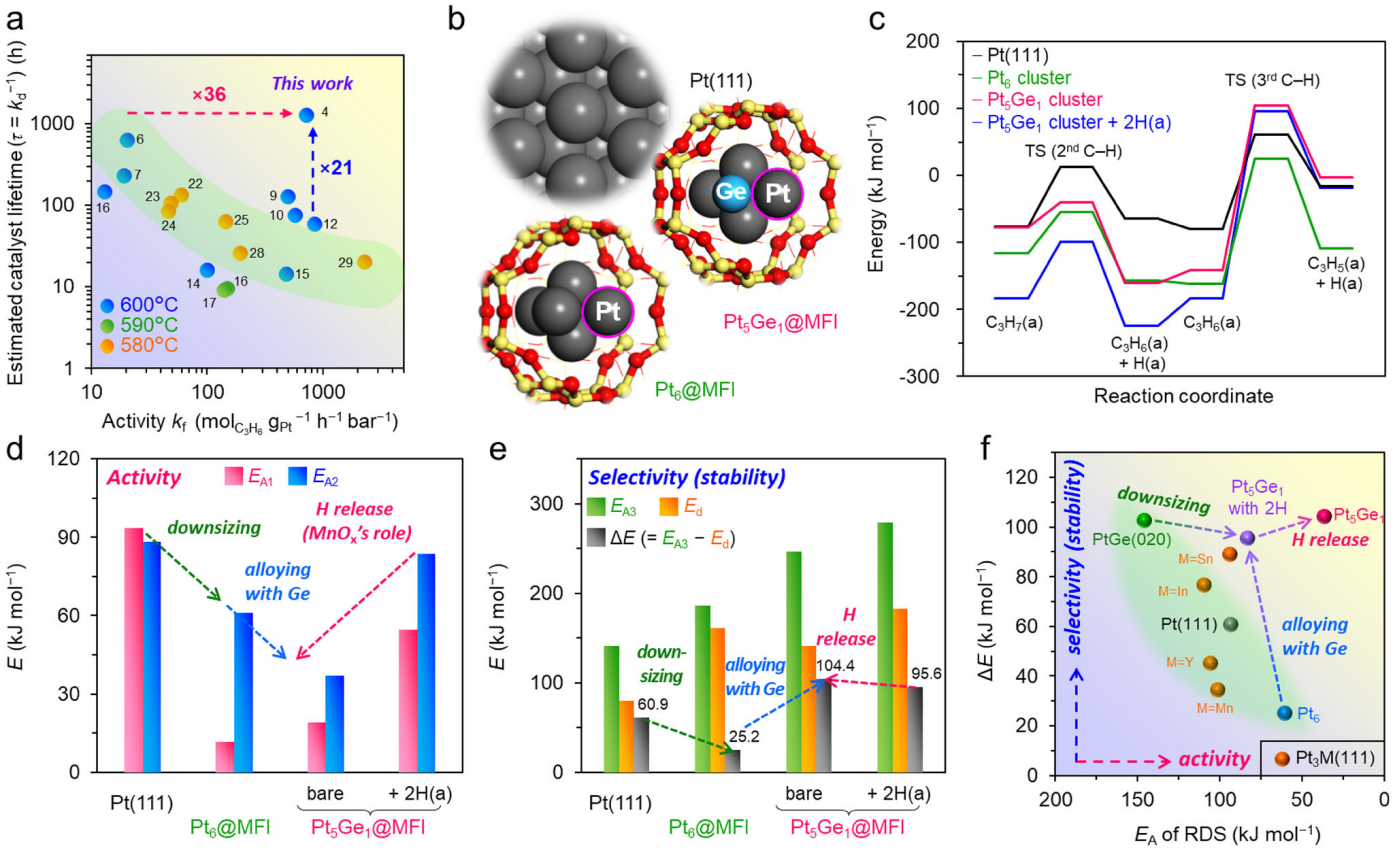
(a) Activity–stability (*k*_f_–*τ*) plot for MnO*_x_*‐PtGe@MFI and reported catalysts in PDH without co‐fed H_2_ (references are listed in Tables S5 and S6). (b) The optimized structure of Pt(111), Pt_6_ cluster, and Pt_5_Ge_1_ alloy cluster. (c) Energy diagrams of PDH on Pt(111), Pt_6_, and Pt_5_Ge_1_ (with and without co‐adsorbed H) calculated by DFT. For simplicity, the first C─H scission was omitted due to the lower energy barrier. Since propane adsorption is physisorption, the energy differences between catalysts are negligible. (d) The energy barriers of the first and second C─H scissions (*E*_A1_ and *E*_A2_). e) The energy barriers of the third C─H scission (*E*_A3_), propylene desorption (*E*_d_), and difference between *E*_A3_ and *E*_d_ (Δ*E* = *E*_A3_ − *E*_d_). (e) Relationship between Δ*E* and *E*_A_ of RDS.

We then tested the catalytic performance of MnO*_x_*‐PtGe@MFI under neat propane (Figure S28). Surprisingly, MnO*_x_*‐PtGe@MFI showed higher catalyst stability (*τ* = 571 h) than that under diluted propane flow. In addition, we tested the catalytic performance under standard conditions at 600°C (without co‐fed hydrogen, Figure 2f). MnO*_x_*‐PtGe@MFI showed a high initial conversion of 60% and underwent a gradual deactivation, which resulted in a 46% conversion even after 30 days. The catalytic activity and stability obtained in the absence of co‐fed H_2_ at various temperatures for MnO*_x_*‐Pt@MFI and the reported PDH catalysts are summarized in Tables S5 and S6. MnO*_x_*‐PtGe@MFI achieved the world‐record for catalyst lifetime (*τ* = 1279 h) and concurrently outstandingly high activity. Figure 3a shows the activity–stability (*k*_f_–*τ*) plot for catalytic performance at standard working temperatures (580°C–600°C). A tradeoff between activity and stability can clearly be observed among the reported systems (including PtSn IMC [40, 43], Pt_3_La IMC [44], and SnO*_x_*‐Pt clusters@MFI [16, 17, 18]), which illustrates the difficulty of solving this dilemma. However, MnO*_x_*‐PtGe@MFI exhibited exceptional performance with 36‐fold higher activity than the most stable catalyst ever reported and twice its stability, and with comparable activity to and 21‐fold higher stability than the most active catalyst (at 600°C). To the best of our knowledge, we report for the first time a PDH catalyst that truly breaks the activity–stability tradeoff under the harsh condition by presenting outstandingly high activity and stability at the same time. The amount of coke deposition on the spent catalyst was quantified using a temperature‐programmed oxidation technique, which revealed that coking was effectively suppressed by Ge incorporation and MnO*_x_* modification (Figure S29 and Table S7). We also conducted an in situ XAFS measurement of MnO*_x_*‐PtGe@MFI under continuous flow of propane to monitor the dynamic behavior of the active site structural changes. No significant changes were observed in the spectra at 600°C (Figure S30) during the tested period, suggesting that reconstruction of the Pt─Ge cluster did not occur. The reusability of the spent MnO*_x_*‐PtGe@MFI catalyst was also confirmed: the catalyst could be reused without loss of the original activity even after a regeneration process (Figure S28).

### 2.4 DFT Calculations for the Origin of High Activity

To determine the origin of the outstanding performance of MnO*_x_*‐PtGe@MFI, we performed density functional theory (DFT) calculations. We considered Pt_6_ and Pt_5_Ge_1_ clusters to be plausible model structures that could fit within the sinusoidal channel of MFI zeolite (<1.0 nm) and were roughly consistent with the experimental *CN*_Pt─Pt_ and *CN*_Pt─Ge_ (Table S2). The optimized structures of Pt_6_ and Pt_5_Ge_1_ are shown in Figures S31 and S32 (see Methods section and Figures S33–S35 for details). For comparison, we also considered the (111) surfaces of Pt and Pt_3_M (M = Sn [5, 6, 7], In [45], Mn [46], and Y [44, 47]), and the (020) surface of PtGe [8], as these have been reported to be active sites of efficient nanoparticulate PDH catalyst. The PDH reaction mechanism has been proposed and accepted as follows: (1) physical adsorption of propane on a Pt site; (2) stepwise (the first and second) C─H scissions to generate propylene (barrier: *E*_A1_ and *E*_A2_); and (3) propylene desorption from the Pt site (*E*_d_). The first or second C─H scission step is typically the rate‐determining step (RDS) [5, 6, 7], which describes the catalytic activity. Furthermore, the third C─H scission of propylene (*E*_A3_) is often considered to be a trigger of undesired side reactions (cracking and coking), which lower propylene selectivity and eventually lead to catalyst deactivation. Therefore, the difference between *E*_A3_ and *E*_d_ (Δ*E* = *E*_A3_ − *E*_d_) has been used as a descriptor for estimating propylene selectivity (and catalyst stability). In this study, we calculated the stepwise C─H scissions from C_3_H_8_ to C_3_H_5_ on model structures (Figure 3b–e; Figures S36–S45 and Tables S8 and S9).

First, we discuss the results regarding the activity (*E*_A1_ and *E*_A2_) (Figure 3c,d; Table S8). For the (111) surface of Pt and Pt_3_M, the *E*_A_ values of RDS ranged within a relatively narrow region (93.6–109.9 kJ mol^−1^), which reflects that the C─H scission of propane on the Pt_3_ hollow sites is structure‐insensitive, as reported for these materials [5, 6, 11] However, the *E*_A_ value of RDS was much higher when a more diluted Pt─Pt ensemble was considered (PtGe(020); *E*_A1_: 146.4 kJ mol^−1^). This result simply reflects the intrinsic difficulty of enhancing PDH activity using an alloy surface. This may be true when the alloy is in a nanoparticulate state. In contrast, the clusters exhibited much lower *E*_A_ values of RDS (*E*_A2_ for Pt_6_ = 61.0 kJ mol^−1^_,_ *E*_A2_ for Pt_5_Ge_1_ = 36.9 kJ mol^−1^). Interestingly, the changes in *E*_A_ followed the Brønsted−Evans−Polanyi (BEP) relationship in all considered structures (Figure S46): i.e., the reaction energy (Δ*E*_r_) of C─H scission (difference between the initial and final states) determines the energy barrier. Since C_3_H_8_ can only undergo physisorption, adsorption energy (*E*_ad_) is less negative and does not significantly depend on the type of active sites (*e*.*g*., Pt(111): −20.1 kJ mol^−1^, Pt_6_: −27.2 kJ mol^−1^). In contrast, C_3_H_7_ and C_3_H_6_ are capable of chemisorption, for which *E*_ad_ values are largely negative and vary greatly (*e*.*g*., from Pt(111): −79.9 to Pt_6_: −161.4 kJ mol^−1^). The quite negative *E*_ad_ of Pt_6_ serves as a driving force to significantly decrease *E*_A_. In terms of electronic states, the *d* electron levels of metal clusters shift upward toward the Fermi level typically when the clusters are highly coordinatively unsaturated [48], which results in greater adsorptivity. Indeed, our DFT calculations confirmed that the *d* electron levels of a Pt atom in Pt_6_ and Pt_5_Ge_1_ were obviously upshifted relative to those of Pt(111) (Figure S34). Thus, downsizing to a subnanometric scale is a promising strategy, not only for maximizing the atom efficiency of Pt but also for the considerable enhancement of activity (turnover frequency) in PDH. When comparing the Pt_6_ and Pt_5_Ge_1_ clusters, Pt_5_Ge_1_ showed lower *E*_A2_ (RDS) than Pt_6_ (Pt_6_: 61.0 kJ mol^−1^ → Pt_5_Ge_1_: 36.9 kJ mol^−1^) despite high similarity in the adsorption conformations (Figure S47). This is due to destabilization of the chemisorbed C_3_H_7_ state (initial state of second C─H scission) upon Ge incorporation (Figure 3c), originating from the lowered *d* states (Figure S34). Therefore, the destabilization of the C_3_H_7_ state by the electronic effect of Ge is the key for enhancing activity. Thus, “downsizing” and “alloying” synergistically contributed to the lowering of the *E*_A_ value of RDS, which enables the outstanding activity.

Then, we investigated the influence of co‐adsorbed hydrogen (H(a)) on the Pt_5_Ge_1_ cluster to follow the experimentally observed *E*_app_ in Figure 2e. Even in the presence of H(a), the TS structure changed little (C─H distance at TS: 1.58 Å [bare] → 1.56 Å [2H(a)]), which suggests that there was no geometric effect of H(a). Conversely, hydrogen adsorption downshifted the *d* electron levels (Figure S48) likely owing to the stabilization of *d* electrons by Pt─H bond formation. Therefore, adding hydrogen on Pt_5_Ge_1_ significantly stabilized the initial state (C_3_H_7_(a)) of the second C─H scission compared with the final state, increasing the *E*_A2_ from 36.9 to 83.6 kJ mol^−1^ (Figure 3c,d; Figures S37–S39). Thus, the co‐adsorbed hydrogen electronically hinders the C─H cleavage step, which is consistent with the increase in *E*_app_ upon hydrogen poisoning. Moreover, the hydrogen adsorption also decreases the number of vacant Pt sites, which likely lowers the preexponential factor of the C─H scission and the resulting rate constant regardless of the *E*_A_ value of RDS. Therefore, reducing the concentration of H(a) on the clusters is essential to achieving the potentially high catalytic activity of Pt_5_Ge_1_, which can be achieved by placing MnO*_x_* in close proximity to the clusters. We also studied the role of MnO*_x_* for hydrogen trap. A simplified Pt/MnO(100) model was employed for qualitative understanding, which revealed that adsorption of two hydrogen atoms onto the MnO moiety was 56.5 kJ mol^−1^ more stable than onto Pt (Figure S49). This strongly suggests that MnO*_x_* acts as a hydrogen trap to decrease its coverage on the Pt─Ge clusters. More detailed accounts on the origins of the high catalytic activity of MnO*_x_*‐PtGe@MFI can be seen in Text S3.

### 2.5 DFT Calculations for the Origin of Long Lifetime

Finally, we discuss the origin of the high propylene selectivity and the resulting long lifetime of the catalyst (Figure 3e). The values of Δ*E*, which is the selectivity descriptor, for the representative models were in the following order: Pt_6_ (25.2 kJ mol^−1^) << Pt(111) (60.9 kJ mol^−1^) < Pt_3_Sn(111) (89.1 kJ mol^−1^) < Pt_5_Ge_1_ (104.4 kJ mol^−1^) (Table S8). The significantly low ∆*E* of Pt_6_ can be attributed to its strong adsorption of propylene (*E*_ad‐propylene_: −164.1 kJ mol^−1^) owing to the high adsorptivity of subnanometric clusters, even though Pt_6_ has a high *E*_A3_ (186.5 kJ mol^−1^). Therefore, downsizing Pt is beneficial to enhancing catalytic activity, but does not enhance selectivity [14], which highlights the activity–selectivity tradeoff in PDH [5, 6, 11, 45]. This tradeoff can also be seen in Figure 3f, in the plot of Δ*E* and *E*_A_ of RDS (activity descriptor). Surprisingly, Pt_5_Ge_1_ showed an exceptionally large ∆*E* despite the largely negative *E*_ad_ value (−141.6 kJ mol^−1^). This is owing to its remarkably high *E*_A3_ (246.1 kJ mol^−1^). Considering the significantly short interatomic distance of Pt─Ge and the resulting distortion of the cluster (Table S2), the third C─H cleavage may be effectively inhibited by the geometric effect of Ge to enhance the selectivity and stability. Thus, alloying with Ge is the key origin of the selectivity (stability) enhancement and overcomes the activity–selectivity (stability) tradeoff (Figure 3e). However, when co‐adsorbed hydrogen is present on Pt_5_Ge_1_, propylene adsorption is strengthened, depending on the number of H(a) species (Figure 3c,e), which resulted in a smaller value of Δ*E*. Therefore, reducing the concentration of H(a) on the clusters by MnO*_x_* is also necessary to achieve the potentially high selectivity (stability) of Pt_5_Ge_1_. Consequently, we can summarize that the combination of (1) subnano‐downsizing, (2) alloying Pt with Ge, and (3) hydrogen trap by MnO*_x_* was the origin of the exceptionally high activity and selectivity (stability) observed for MnO*_x_*‐PtGe@MFI, which more effectively breaks the activity–selectivity (stability) tradeoff (Figure 3a,f).

To follow the theoretical insights, we performed some temperature‐programmed analyses. For propylene desorption, the desorption peak appeared at 24°C for PtGe@MFI (Figure S50), which was much higher than that of PtGe NPs (−20°C), as reported in our previous study [8]. This demonstrates the strong adsorption of propylene onto the Pt─Ge clusters and is consistent with the theoretical result (Figure S34). For surface reaction with propane, catalytic cracking to methane occurred from 400°C on Pt@MFI, whereas it did not occur on PtGe@MFI (Figure S51). This reflects large difference in the capability of side reaction as reflected in *E*_A3_ values (Figure 3c,e). Thus, the greater adsorptivity (activity) by subnano–downsizing and enhanced selectivity (stability) by alloying with Ge were demonstrate not only theoretically, but also experimentally.

## 3 Conclusions

In summary, we designed the Pt─Ge alloy clusters for efficient propane dehydrogenation catalysis. The developed Pt─Ge alloy cluster showed outstandingly high activity, selectivity, and stability. Alloying with Ge, however, caused hydrogen poisoning to lower the catalytic activity. Further modification with MnO*_x_* prevented the hydrogen poisoning, which fully induced the potential ability of PtGe alloy clusters for dehydrogenation. Detailed mechanistic study and theoretical calculations revealed the origin of the exceptionally high catalytic performance as follows: (1) downsizing from nanometric to subnanometric orders is the key for the considerable enhancement of catalytic activity due to the greater adsorptivity on highly coordinatively unsaturated active sites, (2) alloying Pt clusters with Ge changes the electronic environment and geometric cluster distortion, further increasing the catalytic activity and selectivity/stability, respectively, (3) MnO*_x_* dispersion in the vicinity of Pt─Ge alloy clusters prevents hydrogen poisoning, fully inducing their potential ability for dehydrogenation. As a result, the catalyst design concept of combining the “downsizing” and “alloying” functioned well, allowing to break the activity–stability tradeoff in propane dehydrogenation. We believe that this work provides a new dimension for the design of high‐performance catalysts not only for alkane dehydrogenation but also for broader applications.

## Author Contributions

Y.N. and S.F. supervised the study. Y.N. performed most of the experiments and theoretical calculations. Y.N., K.S., and S.F. discussed and revised the paper.

## Conflicts of Interest

The authors declare no Conflicts of Interest.

## Supporting information




**Supporting File**: smll73115‐sup‐0001‐SuppMat.pdf.

## Data Availability

The data that support the findings of this study are available from the corresponding author upon reasonable request.
