# Supplementary material for: Subnanometric Platinum–Germanium Clusters for Efficient Propane Dehydrogenation Catalysis
Source: Small. 2026 Mar 17;22(23):e73115. doi: 10.1002/smll.73115 (PMC13100553; doi:10.1002/smll.73115)
Supplement: Supplementary file 1 — Supporting File: smll73115‐sup‐0001‐SuppMat.pdf. [file SMLL-22-e73115-s001.pdf]

Subnanometric Platinum–Germanium Clusters for Efficient Propane Dehydrogenation Catalysis

Yuki Nakaya[1], Ken-ichi Shimizu[1], Shinya Furukawa[1]*

Experimental details

Materials

KOH (KANTO, ≥86.0%), Tetrapropylammonium Hydroxide (TPAOH, TCI, 20–25 wt%, K-free), Tetraethyl Orthosilicate (TEOS, TCI, ≥97%), $H_2PtCl_4\cdot 6H_2O$ (Wako, ≥99.9%), $(NH_4)_2GeF_6$ (Alfa Aesar, ≥99.99%), $Mn(NO_3)_2\cdot 6H_2O$ (Wako, ≥99.9%), Ethylenediamine (Wako, ≥99%), $SnCl_5\cdot 5H_2O$ (Wako, ≥98%)

One-pot synthesis of Pt-based catalysts encapsulated within the pure silica MFI zeolite.

Synthesis of the as-prepared MnO_x-PtGe@MFI catalyst.

The as-prepared MnO_x-PtGe@MFI was synthesized by a one-pot method according to the previous reports with modifications.[1–3] The addition of K prevents sintering of Pt nanoparticles, but it does not affect the catalytic performance itself. The detailed procedures are as follows.

(1) 8.12 g TPAOH and 4.2474 g TEOS were sequentially added to $KOH_{(aq)}$ (31.0 mg KOH in 5g ion-exchanged water), then the mixture was stirred continuously for 24 h at room temperature.
(2) $(NH_4)_2GeF_{6(aq)}$ (30.5 mg and 0.2 g ion-exchanged water) was added, followed by stirring for 1 h.
(3) A mixed solution (16.0 mg $H_2PtCl_4\cdot 6H_2O$, 39.3 mg $Mn(NO_3)_2\cdot 6H_2O$, and 0.8 g ion-exchanged water) was added, then stirred for 6 h.
(4) 150 μL ethylenediamine was injected into the mixture under vigorous stirring and left to stir for 1 h.
(5) The resultant solution was transferred to a Teflon-lined autoclave and heated in an oven at 175°C for 96 h under static conditions with a ramping rate of 1 °C min^{-1}.
(6) After the hydrothermal process, the resulting solid product was collected by centrifugation using ion-exchanged water.
(7) The collected solid product was washed by filtration using ion-exchanged water (5 times) and acetone (5 times)
(8) The resulting solid was ground into powder, then calcined at 560°C for 8 h in dry air with a ramping rate of 2 °C min^{-1}, then cooled to room temperature.
(9) The powder was further calcined at 600°C for 2 h in dry air with a ramping rate of 5 °C min^{-1}.

Inductively coupled plasma-atomic emission spectroscopy (ICP-AES) revealed that the mass loading of Pt, Ge, and Mn were 0.46 wt%, 0.29 wt%, and 0.40 wt%, respectively, corresponding to the Mn/Ge/Pt molar ratio of 3.3/1.8/1 (Supplementary Table 1). The feed molar ratio of Mn/Ge/Pt was set at 3.0/4.5/1, indicating the loss of Ge component during the synthetic process.

Synthesis of the as-prepared PtGe@MFI catalyst.

The synthesis of the as-prepared PtGe@MFI followed a similar procedure to that of the as-prepared MnO_x-PtGe@MFI except the addition of $Mn(NO_3)_2\cdot 6H_2O$.

Synthesis of the as-prepared MnO_x-Pt@MFIr catalyst.

For the synthesis of MnO_x-Pt@MFI, the step 2 was skipped and a mixed solution of 16.0 mg $H_2PtCl_4\cdot 6H_2O$, 39.3 mg $Mn(NO_3)_2\cdot 6H_2O$, and 1.0 g ion-exchanged water was used for step 3.

Synthesis of the as-prepared Pt@MFI catalyst.

For the synthesis of Pt@MFI, the step 2 was skipped and a mixed solution of 16.0 mg $H_2PtCl_4\cdot 6H_2O$ and 1.0 g ion-exchanged water was used for step 3.

Synthesis of the as-prepared SnO_x-Pt@MFI catalyst.

Instead of $Mn(NO_3)_2\cdot 6H_2O$, $SnCl_5\cdot 5H_2O$ (50 mg) was used as a precursor.

Catalytic reaction

The propane dehydrogenation (PDH) reactions were performed in a vertical, straight-type quartz fixed-bed reactor with an internal diameter of 4 mm under an atmospheric pressure. First, the as-synthesized catalysts were charged in a reactor, subsequently reduced under flowing H_2 (10 mL min^{-1}) at 700°C for 5 h. Then, the reactant gas mixture was fed; C_3H_8:He = 2.5:5.0, a total of 7.5 mL min^{-1}. The detailed amount of each catalyst is given in the corresponding figure's caption. The product gas was analyzed by online gas chromatography equipped downstream with a thermal conductivity detector (TCD; Shimadzu GC–8A) and a column of Gaskuropak 54 (GL Science). For all the catalysts, C_3H_8, C_2H_4, C_2H_6, and CH_4 were detected as reaction products. C_3H_8 conversion, C_3H_6 selectivity, C_3H_8 yield, and material balance were defined by Eqs (1) ~ (4), respectively. Material balance typically ranged between 98~102% for all the reactions.

$$\mathrm{C_3H_8\ conversion\ (\%)} = \frac{[C_3H_8]_{inlet} - [C_3H_8]_{outlet}}{[C_3H_8]_{inlet}} \times 100 \quad (1)$$

$$\mathrm{C_3H_6\ selectivity\ (\%)} = \frac{[C_3H_6]}{[C_3H_6] + \frac{2}{3}[C_2H_6] + \frac{2}{3}[C_2H_4] + \frac{1}{3}[CH_4]} \times 100 \quad (2)$$

$$\mathrm{C_3H_6\ yield\ (\%)} = \frac{[C_3H_6]_{outlet}}{[C_3H_8]_{inlet}} \times 100 \quad (3)$$

$$\mathrm{Material\ balance\ (\%)} = \frac{[C_3H_8]_{outlet} + [C_3H_6] + \frac{2}{3}[C_2H_6] + \frac{2}{3}[C_2H_4] + \frac{1}{3}[CH_4]}{[C_3H_8]_{inlet}} \times 100 \quad (4)$$

To estimate the catalyst stability, the first-order deactivation model was employed.[4] k_d (h^{-1}) and τ (h) were defined by the following equation. k_d (h^{-1})[4] and τ (h) represent the deactivation rate constant and mean catalyst life, respectively. Here, lower k_d and higher τ values represent higher catalyst stability.

$$k_\mathrm{d} = \frac{\ln\left(\frac{1 - conv._{end}}{conv._{end}}\right) - \ln\left(\frac{1 - conv._{start}}{conv._{start}}\right)}{t} \quad (5)$$

$$\tau = \frac{1}{k_d} \quad (6)$$

where, $conv._\mathrm{start}$ and $conv._\mathrm{end}$ indicates the initial and final C_3H_8 conversion, respectively. t indicates time on stream.

Besides, the rate constant for the forward direction (k_f) of PDH was also estimated to evaluate the catalytic activity using equation 7.

$$k_\mathrm{f} = \frac{R}{P_{\mathrm{C_3H_8}}\left(1 - \frac{1}{K_e}\frac{P_{\mathrm{C_3H_6}}P_{\mathrm{H_2}}}{P_{\mathrm{C_3H_8}}}\right)} \quad (7)$$

where, R, P_X, and K_e are the specific activity [$\mathrm{mol_{C3H6}\ g_{Pt}^{-1}\ h^{-1}}$], partial pressure of X [bar], and equilibrium constant [bar].[5,6]

Characterization

The crystalline phase of the MFI zeolite in the prepared catalysts was characterized using powder X-ray diffraction (XRD, Rigaku, MiniFlex 700+D/teX Ultra; Cu Kα X–ray source).

The actual metal contents were measured using inductively coupled plasma atomic emission spectroscopy (ICP-AES) at the Instrumental Analysis Division, Global Facility Center, Creative Research Institution, Hokkaido University. Each catalyst was dissolved in the combined aqueous solution of hydrofluoric acid, hydrochloric acid, and nitric acid before the analysis.

High angle annular dark field scanning transmission electron microscopy (HAADF-STEM) was used to confirm the particle size distribution and the crystal structure using a FEI Titan G2 microscope equipped with an energy dispersive X-ray (EDX) analyzer operated at 300 kV. In addition, integrated differential phase contrast (iDPC) was conducted to observe the framework of MFI zeolite using a FEI Titan G2 microscope. Prior to the observation, a catalyst was firstly ground and dispersed in ethanol (Wako, ≥99.5%) by ultrasonic. Then, the dispersed catalyst was deposited on a molybdenum or cupper grid and dried in vacuum. The particle size distribution was estimated by using volume mean particle size.

The amount of coke accumulated on a spent catalyst was evaluated by temperature-programmed oxidation (TPO). First, 50 mg of the fresh catalyst was used for PDH at 600°C for 19.5 h. Then, the spent catalyst was transferred into a quartz tube reactor. Prior to the measurement, the catalyst was first pretreated at 150°C for 1 h under flowing He (20 mL min^{-1}), and then cooled to 100°C, subsequently heated from 100°C to 700°C with a ramping rate of 2 °C min^{-1} under flowing 2% O_2/He (50 mL min^{-1}). The outlet CO_2 (m/z = 44) was analyzed online by quadrupole mass spectrometer (BELMASS) equipped downstream.

X-ray absorption fine structure (XAFS) spectra (Pt L_{III}-, Pt L_{II}-, Ge K-, Mn K-edges) were performed at BL01B1 and BL14B2 beamlines of SPring-8, JASRI, a Si(111) double-crystal monochromator. Prior to the measurement, the pelletized sample (diameter of 7 mm, *ca*. 40 mg) of as-calcined (not reduced) catalyst was transferred into a quartz cell and reduced at 700°C for 60 min under flowing 50% H_2/He (50 mL min^{-1}), and then cooled to room temperature under flowing He (25 mL min^{-1}). After the pretreatment, XAFS spectra was recorded in fluorescence mode at room temperature under He flow. The referential foil and oxide samples were measured in transmission mode at room temperature. The obtained XAFS spectra were analyzed using Athena and Artemis software ver. 0.9.25 implemented in the Demeter package.[7] The back-scattering amplitude and phase shift functions were calculated by FEFF8.[8] *R*-factor (R^2) for curve-fitting was defined as follows: $R^2 = \Sigma i\{k^3\chi_i^{exp}(k)-k^3\chi_i^{fit}(k)\}^2/\Sigma i\{k^3\chi_i^{exp}(k)\}.^2$ As shown in Supplementary Fig. 7, Pt L_{III}-edge XAFS spectra was interfered by Ge K-edge oscillation. Besides, Pt L_{II}-edge XANES spectra is more sensitive to the formation of Pt-based alloys compared with Pt L_{III}-edge XANES spectra (Fig. 1d and Supplementary Fig. 7). Importantly, *CN* values in Pt L_{III}- and Pt L_{II}-edges were similar in Pt@MFI and MnOx-Pt@MFI (Supplementary Table 2). Therefore, Pt L_{II}-edge EXAFS curve fitting is adequate for analyzing the local structures of Pt-based catalysts. Therefore, we mainly focused on Pt L_{II}-edge XAFS spectra in this study. The XAFS spectrum of bulk PtGe was reproduced by our previous report.[9] The bulk PtGe showed a diffraction pattern characteristic to intermetallic PtGe.

Wavelet transformation of the experimental k^3-weighted EXAFS spectra was performed using HAMA Fortran code.[10–12] A Cauchy wavelet of order 200 was used due to the balance between a reasonable *k*- and *R*-space resolutions.[13]

C_3H_6 temperature-programed desorption (C_3H_6-TPD) experiments were conducted using a BELCAT-II (Microtrac BEL) instrument. Prior to C_3H_6 adsorption, the unreduced catalyst was reduced at 700°C for 5 h under a flow of 5% H_2/Ar. Following reduction, the catalyst was cooled to −35 °C under an argon Ar flow using a CATCryo-II unit, subsequently the catalyst was exposed to a 5% C_3H_6/He gas mixture. Physisorbed C_3H_6 was removed by purging with helium at −35°C. The catalyst was then heated from −35°C to 150°C at a ramping rate of 2 °C min^{-1}. The desorbed C_3H_6 (m/z = 41) was monitored by a downstream quadrupole mass spectrometer (BELMASS). A second series of C_3H_6-TPD experiments, ranging from 100°C to 600°C, was performed using a similar methodology. For these experiments, 100%H_2, 100%C_3H_6, and argon were used as the reduction, adsorption, and purge gases, respectively. The heating rate for this series was set at 1 °C min^{-1}.

DFT calculations

Periodic Density Functional Theory (DFT) calculations were carried out using the Cambridge Sequential Total Energy Package (CASTEP) code[14] with Vanderbilt-type ultrasoft pseudopotentials and the revised version of Perdew−Burke−Ernzerhof exchange−correlation functional based on the generalized gradient approximation.[15] The plane-wave basis set was with a cut-off energy of 600 eV was assigned. A Fermi smearing of 0.1 eV was used. Dispersion correlations were considered using the Tkatchenko–Scheffler method with a scaling coefficient of $s_R = 0.94$ and a damping parameter of $d = 20$.[16] The reciprocal space was sampled using a k-point mesh with a spacing of typically 0.04 Å^{-1}, as generated by the Monkhorst−Pack scheme.[17] Geometry optimizations were performed using periodic boundary conditions. All atoms were allowed to fully relax during calculations. The convergence criteria for structure optimization and energy calculation were set to (a) an self-consistence field (SCF) tolerance of 1.0×10^{-6} eV per atom, (b) an energy tolerance of 1.0×10^{-5} eV per atom, (c) a maximum force tolerance of 0.05 eV Å^{-1}, and (d) a maximum displacement tolerance of 1.0×10^{-3} Å. For all calculations, the net charge was set to zero and spin polarization was considered.

The unit cell size of the pure silica MFI was first optimized. Then, Pt_6 cluster was constructed inside of a sinusoidal channel, followed by optimization with the size of MFI fixed. Among three Pt_6@MFI structures calculated, the most stable structure was chosen for latter calculation. A Pt atom of Pt_6@MFI was replaced with Ge, then optimized with the size of MFI fixed. The most stable Pt_5Ge_1@MFI was selected as a model for calculation among six structures. For the referential surface models (Pt(111), Pt_3M(111) (M = Sn, In, Mn, Y), PtGe(020), and MnO(200) a thickness of six atomic layers with 13 Å of vacuum spacing was used. The unit cell size of the bulk crystal was first optimized, followed by modeling the slab structure and surface relaxation with the size of the supercell fixed. The adsorption energy was defined as follows: $E_{ad} = E_{A\text{-}S} - (E_S + E_A)$, where $E_{A\text{-}S}$ is the energy of the slab together with the adsorbate, E_A is the total energy of the free adsorbate, and E_S is the total energy of the bare slab. For the Pt/MnO surface, the Pt_5 cluster was placed on the MnO(200) model, followed by structural optimization. Transition state search was carried out based on the complete linear synchronous transit/quadratic synchronous transit method[18,19] with the tolerance for all root-mean-square forces on an atom of 0.10 eV Å^{-1}.

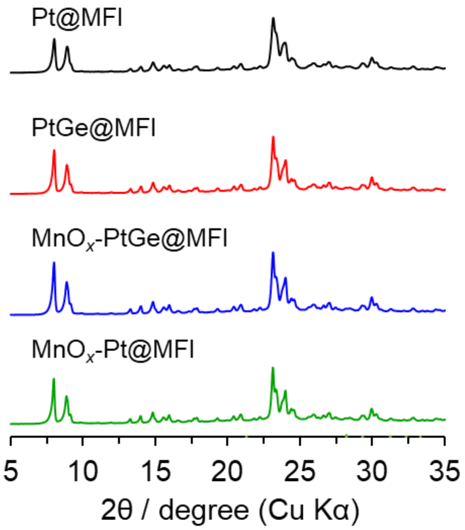

Figure S1. XRD patterns of the as-prepared samples of Pt@MFI, PtGe@MFI, MnO_x-PtGe@MFI (Mn/Ge/Pt = 3.3/1.8/1), and MnO_x-Pt@MFI. All catalysts show the similar XRD patterns, which well matched with pure silica MFI structure.[1,2]

Table S1. Summary of loading amounts and composition ratios of pure silica MFI-supported Pt-based catalysts.

Catalyst (as-prepared)	loading amount (wt%)					Molar ratio			
	K	Pt	Ge	Mn	Sn	K/Pt	Ge/Pt	Mn/Pt	Sn/Pt
Pt@MFI	0.55	0.43	–	–	–	3.2	–	–	–
PtGe@MFI	0.61	0.46	0.28	–	–	3.3	1.6	–	–
MnO_x-PtGe@MFI-S	0.63	0.46	0.25	0.20	–	3.4	1.5	1.5	–
MnO_x-PtGe@MFI	0.60	0.43	0.29	0.40	–	3.5	1.8	3.3	–
MnO_x-PtGe@MFI-L	0.59	0.39	0.28	0.59	–	3.8	1.9	5.4	–
MnO_x-Pt@MFI	0.55	0.40	–	0.57	–	3.4	–	5.1	–
SnO_x-Pt@MFI	0.87	0.40	–	–	1.04	5.4	–	–	14.3

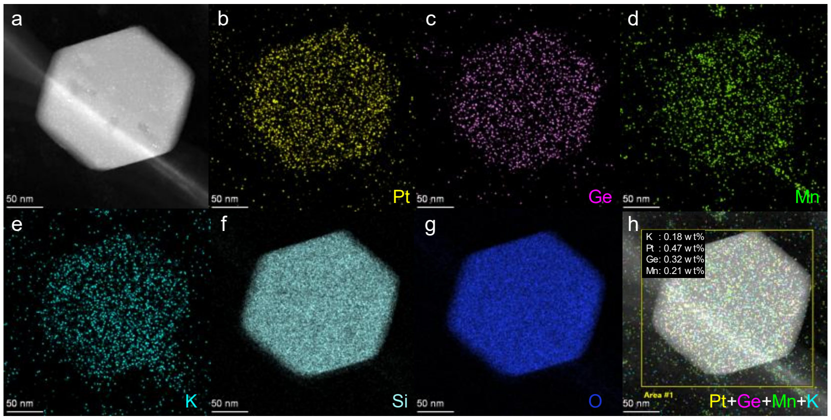

Figure S2. (a) HAADF-STEM image of the MnO_x-PtGe@MFI catalyst (Mn/Ge/Pt = 3.3/1.8/1). Elemental maps of (b) Pt, (c) Ge, (d) Mn, (e) K, (f) Si, (g) O, and (h) HAADF+Pt+Ge+Mn+K overlap acquired using EDX. The composition ratio shown in (h) is roughly consistent with the result obtained by ICP-AES (Supplementary Table 1).

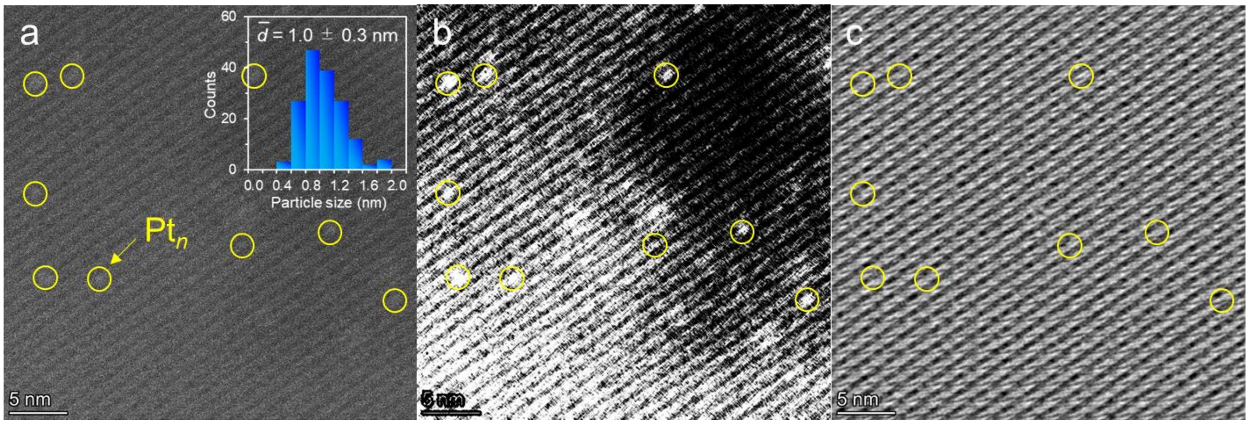

Figure S3. (**a**) HAADF-STEM, (**b**) modified HAADF-STEM (drawn line mode), and (**c**) corresponding iDPC images of the MnO_x-PtGe@MFI catalyst (Mn/Ge/Pt = 3.3/1.8/1). Inset shows (**a**) particle size distribution of Pt_n clusters.

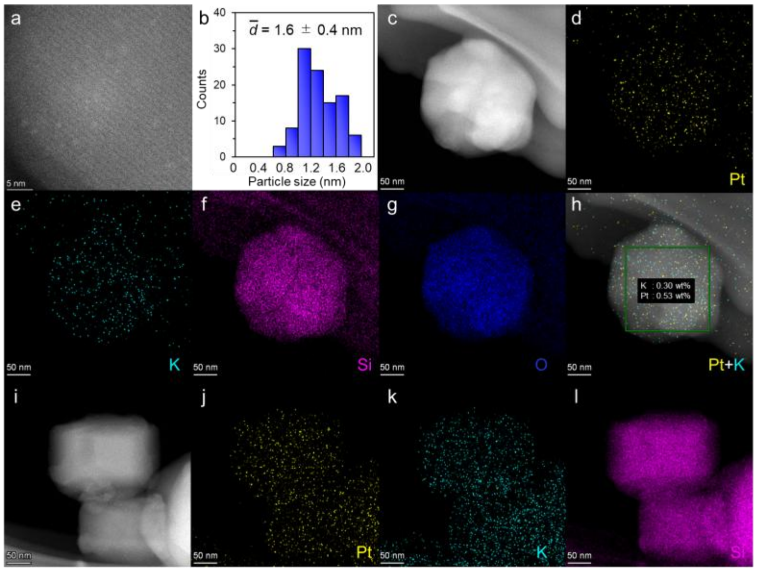

Figure S4. (**a**) HAADF-STEM of the Pt@MFI catalyst. (**b**) Particle size distribution for more than 100 nanoparticles. (**c**) HAADF-STEM and corresponding elemental maps of (**d**) Pt, (**e**) K, (**f**) Si, (**g**) O, and (**h**) HAADF+Pt+K overlap acquired using EDX. The loading of Pt and K shown in (h) is roughly consistent with the result obtained by ICP-AES (Supplementary Table 1). (**i**) HAADF-STEM and corresponding elemental maps of (**j**) Pt, (**k**) K, (**l**) Si.

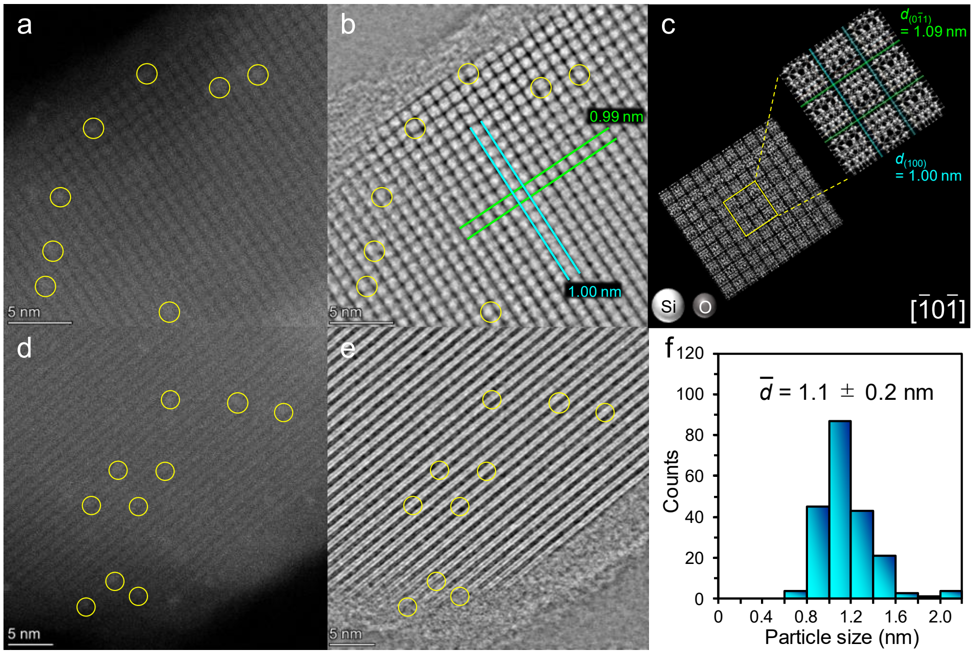

Figure S5. (**a**) HAADF-STEM and (**b**) corresponding iDPC images of the PtGe@MFI catalyst. (**c**) Pure silica MFI structure viewed along $[\bar{1}0\bar{1}]$ direction. (**d**) HAADF-STEM and (**e**) corresponding iDPC images of the PtGe@MFI catalyst. (**f**) Particle size distribution for more than 200 nanoparticles. Subnanometric Pt-based clusters (bright contrast) are emphasized as the yellow circles in (**a**), (**b**), (**d**), and (**e**). HAADF-STEM images revealed the presence of subnanometric Pt-based clusters. Importantly, the corresponding location of subnanometric Pt-based clusters in iDPC images overlapped with MFI framework. These results indicates that the subnanometric Pt-based clusters were regeoselectively encapsulated with in the sinusoidal channels of pure silica MFI, as seen in MnO_x-PtGe@MFI (Main manuscript).

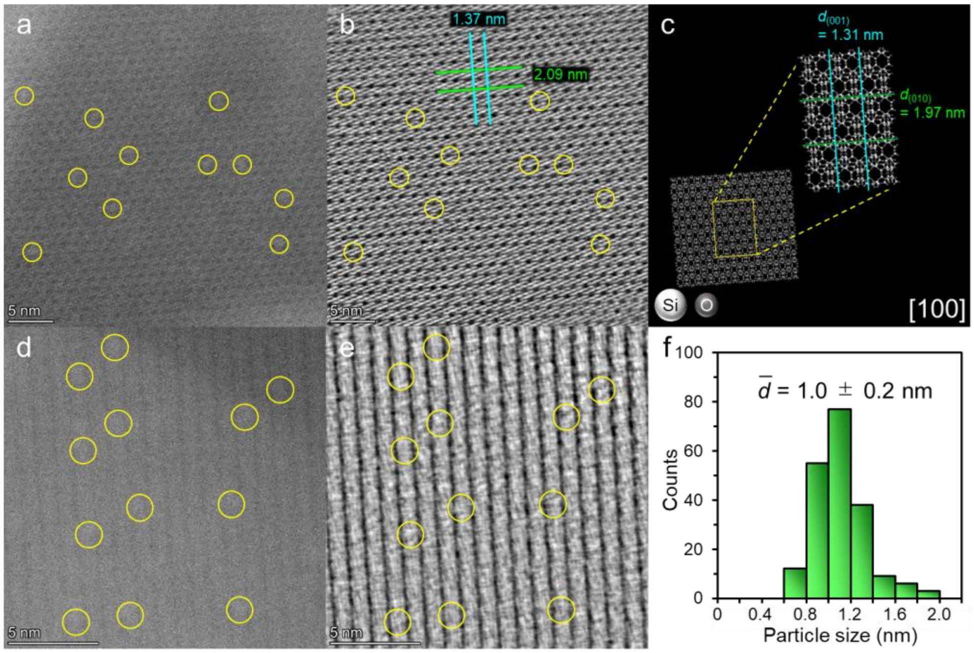

Figure S6. (**a**) HAADF-STEM and (**b**) corresponding iDPC images of the SnO_x-Pt@MFI catalyst. (**c**) Pure silica MFI structure viewed along $[\bar{1}0\bar{1}]$ direction. (**d**) HAADF-STEM and (**e**) corresponding iDPC images of the SnO_x-Pt@MFI catalyst. (**f**) Particle size distribution for more than 200 nanoparticles.

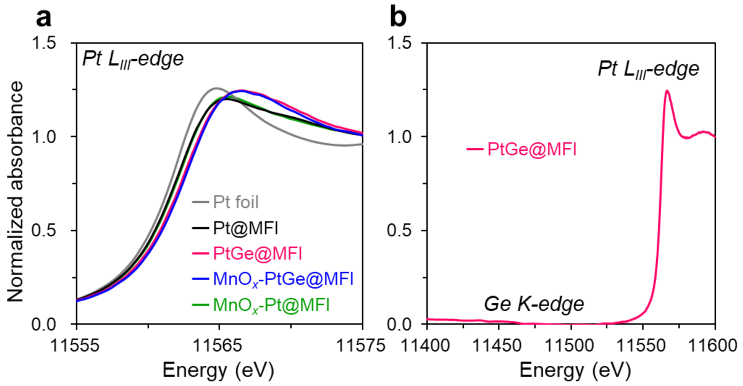

Figure S7. (a) Pt L_{III}-edge XANES spectra of Pt foil, Pt@MFI, PtGe@MFI, MnO_x-PtGe@MFI, MnO_x-Pt@MFI. Reduction at 700°C for 5 h (Pt@MFI: 700°C for 1 h). (b) Pt L_{III}-edge XANES spectrum of PtGe@MFI. For Ge-containing Pt-based catalysts, Pt L_{III}-edge XAFS spectra was interfered by Ge K-edge oscillation.

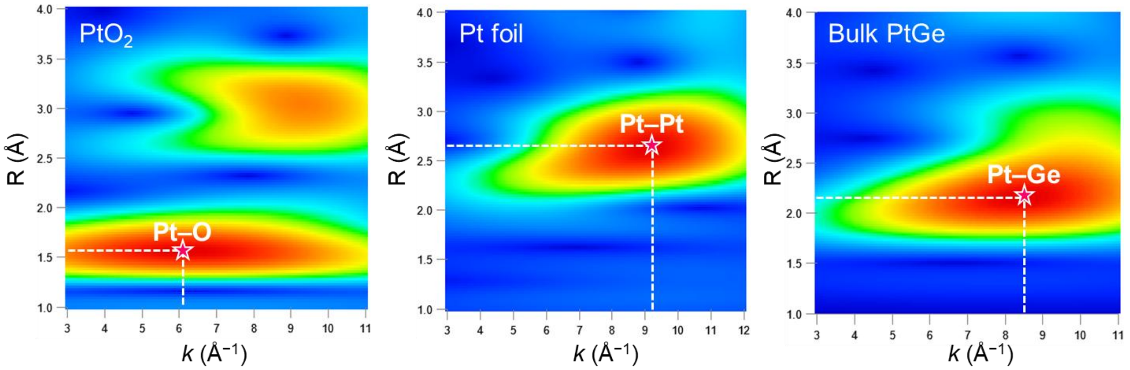

Figure S8. Wavelet transformed Pt L_{II}-edge XAFS signals of the PtO_2, Pt foil, and bulk PtGe.

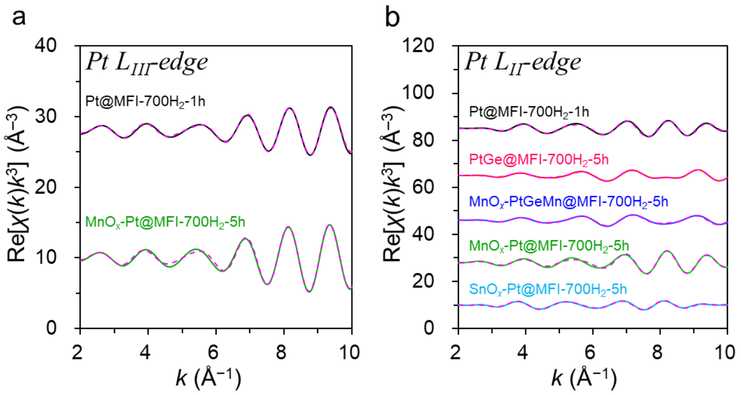

Figure S9. (a) Pt L_{III}-edge k^3-weighted raw EXAFS oscillations of the Pt@MFI and MnO$_x$-Pt@MFI catalysts. Corresponding curve-fitting results of the k^3-weighted EXAFS oscillations (dashed pink lines) are overlapped with the raw oscillations. The fitting ranges are as follows: Pt@MFI; Δk = 3−13 Å^{-1} and Δr = 2.0−3.2 Å. MnO$_x$-Pt@MFI; Δk = 3−12 Å^{-1} and Δr = 2.0−3.2 Å. (b) Pt L_{II}-edge k^3-weighted raw EXAFS oscillations of the Pt@MFI, PtGe@MFI, MnO$_x$-PtGe@MFI, MnO$_x$-Pt@MFI, and SnO$_x$-Pt@MFI catalysts. Corresponding curve-fitting results of the k^3-weighted EXAFS oscillations (dashed pink lines) are overlapped with the raw oscillations. The fitting ranges are as follows: Pt@MFI; Δk = 3−10 Å^{-1} and Δr = 2.0−3.4 Å. PtGe@MFI; Δk = 3−10 Å^{-1} and Δr = 1.3−3.7 Å. MnO$_x$-PtGe@MFI; Δk = 3−10 Å^{-1} and Δr = 1.3−3.7 Å. MnO$_x$-Pt@MFI; Δk = 3−10 Å^{-1} and Δr = 1.8−3.4 Å. SnO$_x$-Pt@MFI; Δk = 3−9 Å^{-1} and Δr = 1.3−3.7 Å.

Table S2. Summary of the Pt L_{III}- and Pt L_{II}-edges k^3-weighted EXAFS curve fittings.

Sample	Edge	Shell	S_0^2[a]	CN[b]	R (Å)[c]	ΔE_0 (eV)[d]	σ^2 (Å^2)[e]	R-factor (R^2)
Pt L_{III}-edge								
Pt foil	Pt L_{III}	Pt–Pt	0.93	12.0 (fix)	2.77 ± 0.00	2.2 ± 0.5	0.005 ± 0.000	0.001
Pt@MFI[f]	Pt L_{III}	Pt–Pt	0.93	6.4 ± 0.9	2.72 ± 0.01	0.1 ± 1.7	0.009 ± 0.001	0.008
MnO$_x$-Pt@MFI[f]	Pt L_{III}	Pt–Pt	0.93	7.1 ± 1.2	2.73 ± 0.01	−1.4 ± 2.1	0.007 ± 0.001	0.008
Ge K- and Pt L_{II}-edges								
GeO$_2$	Ge K	Ge–O	1.03	4.0 (fix)	1.75 ± 0.01	5.9 ± 1.9	0.003 ± 0.001	0.005
Pt foil	Pt L_{II}	Pt–Pt	0.96	12.0 (fix)	2.78 ± 0.01	4.4 ± 1.1	0.005 ± 0.000	0.002
Bulk PtGe	Pt L_{II}	Pt–Pt	0.96	4.7 ± 0.9	2.54 ± 0.01	0.6 ± 2.3	0.009 ± 0.001	0.006
		Pt–Ge	0.96	4.3 ± 1.8	2.93 ± 0.01		0.008 ± 0.003	
Pt@MFI[f]	Pt L_{II}	Pt–Pt	0.96	6.9 ± 1.7	2.73 ± 0.01	5.0 ± 2.0	0.011 ± 0.002	0.009
PtGe@MFI[f]	Pt L_{II}	Pt–Ge	0.96	1.4 ± 0.6	2.40 ± 0.02	−2.2 ± 2.3	0.005 ± 0.003	0.015
		Pt–Pt	0.96	5.4 ± 1.7	2.72 ± 0.02		0.010 ± 0.003	
	Ge K	Ge–O	1.03	3.6 ± 0.8	1.82 ± 0.02	5.8 ± 3.6	0.008 ± 0.003	0.018
		Ge–Pt	1.03	1.4 (fix)	2.50 ± 0.03		0.010 ± 0.003	
MnO$_x$-PtGe@MFI[g]	Pt L_{II}	Pt–Ge	0.96	1.1 ± 0.8	2.39 ± 0.02	−4.6 ± 3.0	0.004 ± 0.004	0.023
(Mn/Ge/Pt = 1.5/1.5/1)		Pt–Pt	0.96	8.6 ± 3.1	2.69 ± 0.03		0.017 ± 0.003	
	Ge K	Ge–O	1.03	3.5 ± 0.9	1.78 ± 0.02	4.0 ± 4.0	0.008 ± 0.003	0.019
		Ge–Pt	1.03	1.2 (fix)	2.48 ± 0.02		0.006 ± 0.003	
MnO$_x$-Pt@MFI[g]	Pt L_{II}	Pt–Pt	0.96	6.7 ± 2.0	2.73 ± 0.02	2.9 ± 2.9	0.007 ± 0.002	0.026
SnO$_x$-Pt@MFI[g]	Pt L_{II}	Pt–O	0.96	1.4 ± 1.2	2.71 ± 0.06	−1.4 ± 3.6	0.003 ± 0.016	0.009
		Pt–Pt	0.96	7.9 ± 3.0	2.72 ± 0.04		0.015 ± 0.003	

[a]Amplitude factor. [b]Coordination number. [c]Distance between absorber and backscatterer atoms. [d]Correction term in the absorption edge. [e]Disorder term (EXAFS Debye–Waller factor). [f]Reduced at 700°C for 1 h.[g]Reduced at 700°C for 5 h.

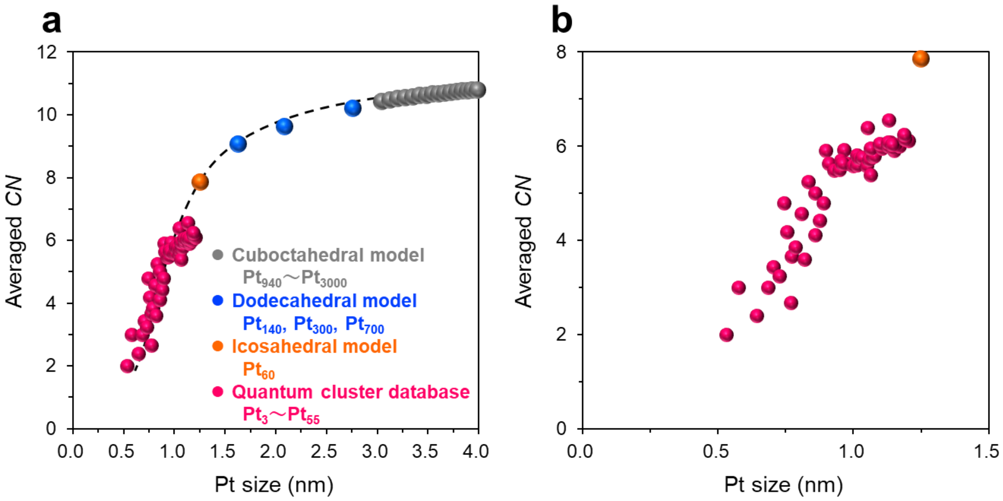

Figure S10. First shell Pt–Pt *CN* as a function of Pt size. This trend is well consistent with the reports by Koningsberger *et al*.[20]

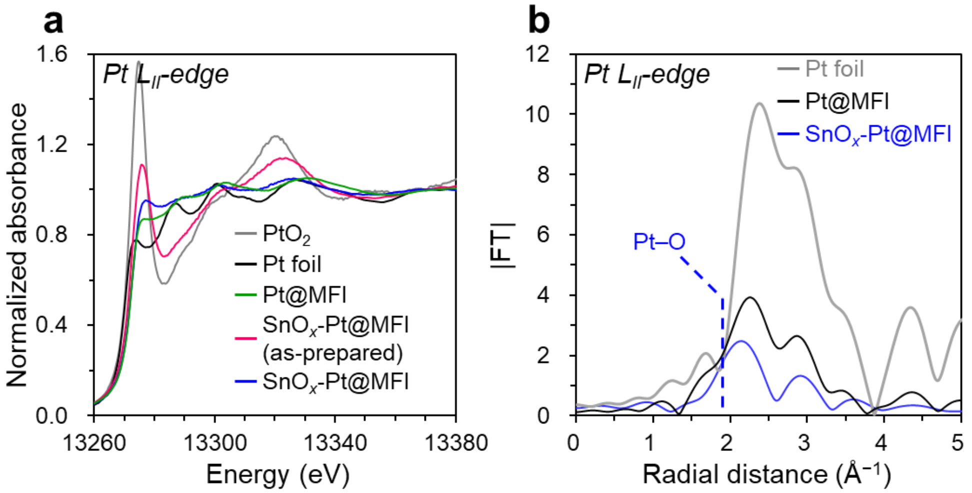

Figure S11. (a) Pt L_{II}-edge XANES spectra of PtO_2, Pt foil, Pt@MFI (Reduction: 700°C for 1 h), SnO_x-Pt@MFI (as-prepared), SnO_x-Pt@MFI (Reduction: 700°C for 5 h). (b) Pt L_{II}-edge FT-EXAFS spectra of Pt foil, Pt@MFI, SnO_x-PtGe@MFI.

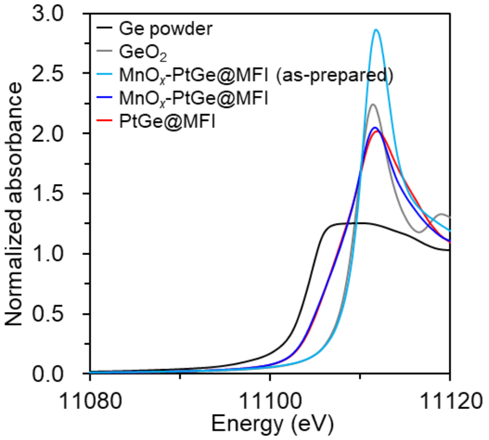

Figure S12. Ge-K edge XANES spectra of the references and MnO_x-PtGe@MFI (as-prepared), MnO_x-PtGe@MFI, and PtGe@MFI catalysts

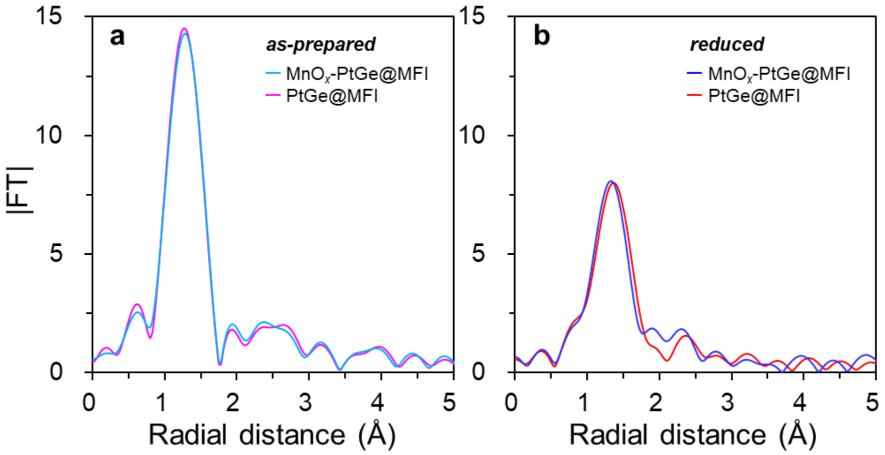

Figure S13. (**a**) Ge-K edge Fourier-transformed EXAFS spectra of the MnO_x-PtGe@MFI (as-prepared) and PtGe@MFI (as-prepared) samples. (**b**) Ge-K edge Fourier-transformed EXAFS spectra of the MnO_x-PtGe@MFI and PtGe@MFI catalysts. Both samples showed similar spectra before and after reduction.

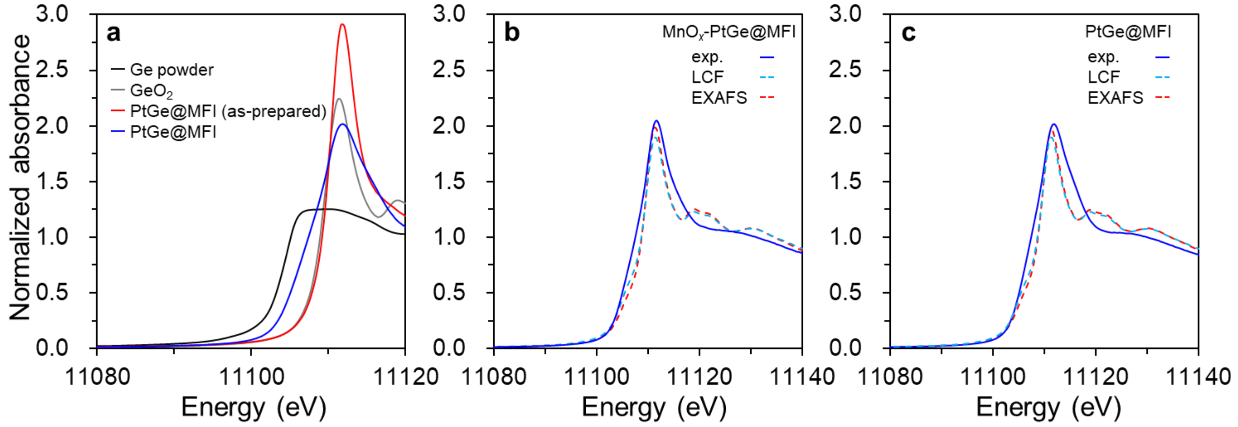

Figure S14. (**a**) Ge-K edge XANES spectra of the Ge powder, GeO_2, PtGe@MFI (as-prepared), and PtGe@MFI. Ge K-edge XANES spectra estimated by using LCF fitting and EXAFS curve fitting results for the (**b**) MnO_x-PtGe@MFI and (**c**) PtGe@MFI catalysts, respectively. The detailed component ratios estimated by LCF and EXAFS curve fittings are shown in Supplementary Table 3.

Table S3. Summary of the LCF fitting results for Ge K-edge XANES spectra.

Sample	Calculation method	Estimated percentage (%)	
		GeO_2	Ge powder
MnO_x-PtGe@MFI	LCF fitting	65.1	34.9
	EXAFS curve fitting	74.6	25.4
PtGe@MFI	LCF fitting	65.4	34.6
	EXAFS curve fitting	72.1	27.9

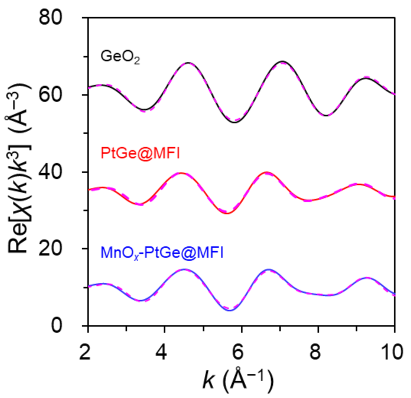

Figure S15. Ge K-edge k^3-weighted raw EXAFS oscillations of GeO_2, PtGe@MFI, and MnO_x-PtGe@MFI. Corresponding curve-fitting results of the k^3-weighted EXAFS oscillations (dashed pink lines) are overlapped with the raw oscillations. The fitting ranges are as follows: GeO_2; Δk = 3−10 Å^{-1} and Δr = 0.8−2.2 Å. PtGe@MFI and MnO_x-PtGe@MFI; Δk = 3−10.5 Å^{-1} and Δr = 0.8−2.5 Å.

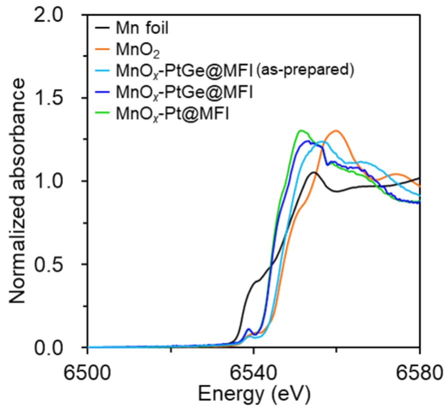

Figure S16. Mn K-edge XANES spectra of the references and MnO_x-PtGe@MFI (as-prepared), MnO_x-PtGe@MFI, and MnO_x-Pt@MFI catalysts.

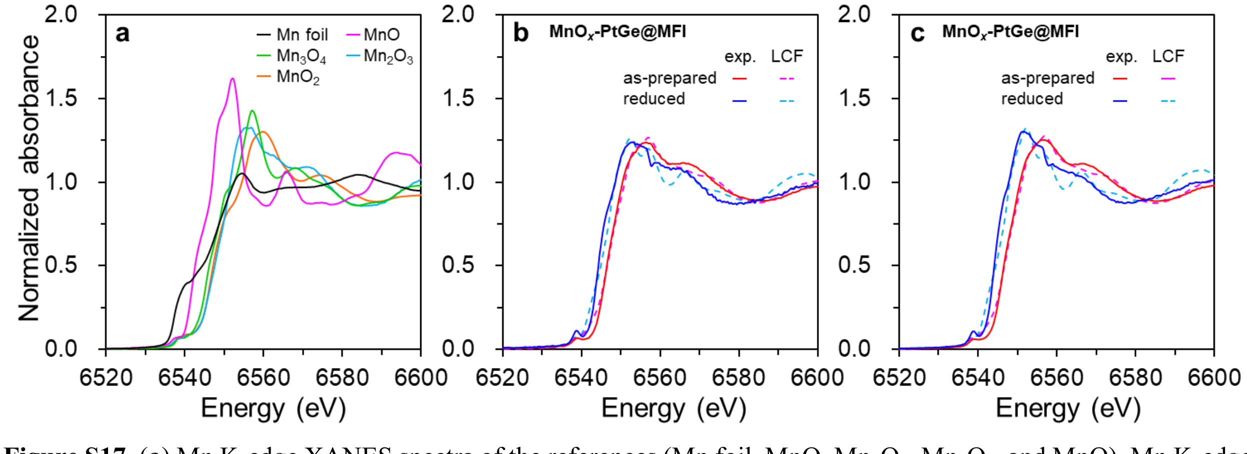

Figure S17. (**a**) Mn K-edge XANES spectra of the references (Mn foil, MnO, Mn_3O_4, Mn_2O_3, and MnO). Mn K-edge XANES spectra of the (**b**) MnO_x-PtGe@MFI (Mn/Ge/Pt = 1.5/1.5/1) and (**c**) MnO_x-Pt@MFI catalysts. Solid and dashed lines indicate the experiment and LCF results, respectively. The detailed component ratios estimated by LCF are shown in Supplementary Table 4. The LCF well reproduced the experiment data, which indicate the accuracy of the obtained LCF fittings.

Table S4. Summary of the LCF fitting results for Mn K-edge XANES spectra.

Sample	Estimated percentage by LCF (%)			
	MnO_2	Mn_2O_3	Mn_3O_4	MnO
MnO_x-PtGe@MFI (as-prepared)	15.1	56.5	16.2	12.2
MnO_x-PtGe@MFI	0	0	47.0	53.0
MnO_x-Pt@MFI (as-prepared)	16.0	51.6	17.2	15.2
MnO_x-Pt@MFI	4.8	0	51.4	43.8

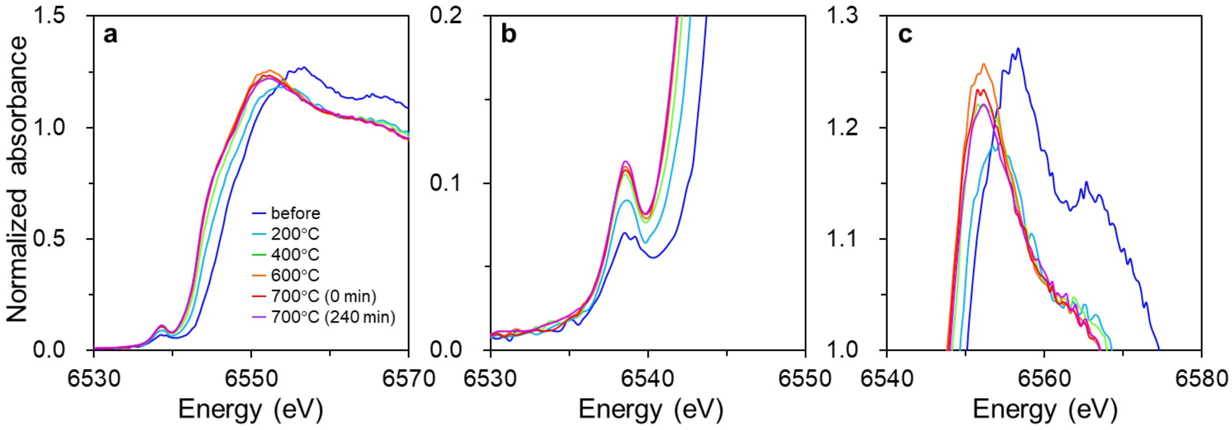

Figure S18. (a,b,c)*In-situ* Mn-K edge XANES spectra of MnO_x-PtGe@MFI (as-prepared). MnO_x-PtGeMn@MFI (as-prepared) sample (Mn/Ge/Pt = 3.3/1.8/1) was utilized as a starting material, and recorded under flowing 50%H_2/N_2 (50 mL min^{-1}). Figures b–c are magnified views of (a).

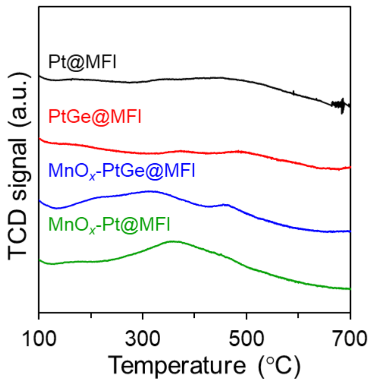

Figure S19. H_2-TPR profiles. The as-prepared samples of Pt@MFI, PtGe@MFI, MnO_x-PtGe@MFI, and MnO_x-Pt@MFI were used as starting materials.

Supplementary text 1. In propane dehydrogenation, the generated propylene acts as a trigger for side reactions, ultimately leading to coke formation. Therefore, if we broadly categorize propane conversion into high and low regions, the former represents an environment where catalyst deactivation is more pronounced due to the higher propylene concentration, while the latter, deactivation is suppressed due to the lower propylene concentration. Indeed, numerous studies have reported that conversion typically stabilizes between 5% and 10% after catalyst deactivation.[1,21,22] Given that our less durable Pt@MFI and MnO_x-Pt@MFI catalysts exhibited stable conversions of around 10% after deactivation, it is reasonable to expect that other catalysts will also eventually stabilize at approximately 10% conversion, rather than dropping to 0%.

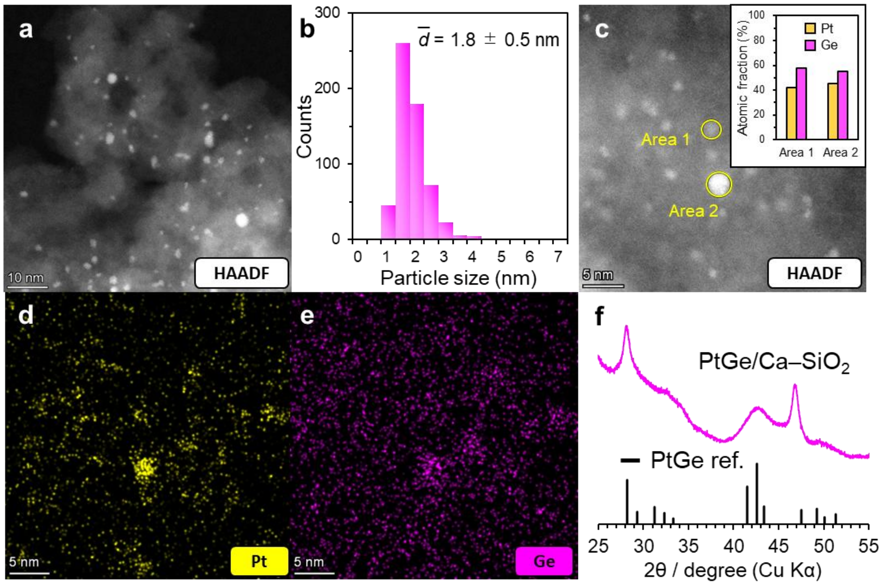

Figure S20. Structural analysis of PtGe/Ca–SiO_2 catalyst (PtGe IMC: Pt 1 wt%) reproduced with permission from ref[9] with slight modification. Copyright 2022 American Chemical Society. (a) HAADF-STEM image and (b) the particle size distribution for 1000 nanoparticles. (c) HAADF-STEM image. Inset bar chart in (c) indicates the atom% included in yellow circles. The ratios of Ge/Pt in the nanoparticles were ca. 1, which is consistent with the ideal ratio of intermetallic PtGe. Elemental maps of (d) Pt and (e) Ge acquired using EDX for the region designated in (c). (f) XRD patterns.

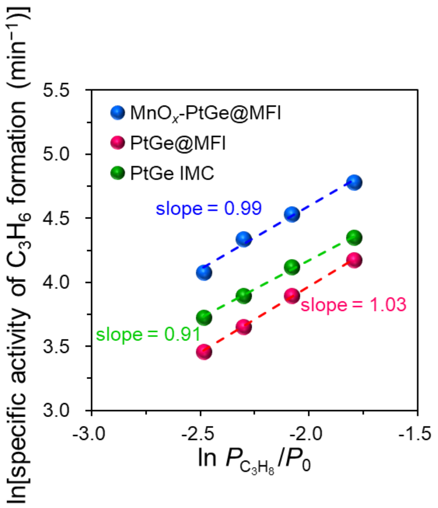

Figure S21. Propane partial dependence for propane dehydrogenation on PtGe@MFI, MnO$_x$-PtGe@MFI (Mn/Ge/Pt = 3.3/1.8/1), and PtGe IMC nanoparticle (PtGe/SiO$_2$, Pt 1wt%, Ge/Pt = 2). Conditions: $C_3H_8/H_2/He$ = 2.5/0.1x/(0.9x)−2.5), $F = x$ mL min^{-1} (x = 10, 15, 20, and 25). Temperature was set at 570°C. Reaction orders of C_3H_8 pressure on catalytic activities in all catalysts showed similar values to 1, indicating the 1st or 2nd C–H scission of propane is the rate-determining step.

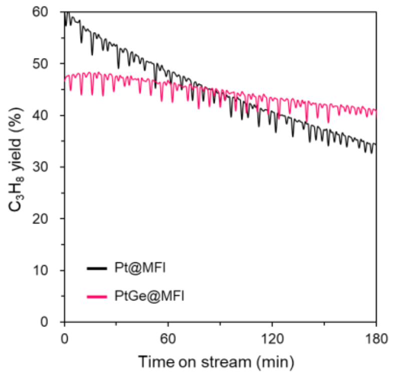

Figure S22. C_3H_8 yield of the Pt@MFI (5 mg) and PtGe@MFI (60 mg) catalyst at 300°C. Conditions: $C_3H_6/H_2/Ar$ = 2.5/2.5/47.5, F = 52.5 mL min^{-1}.

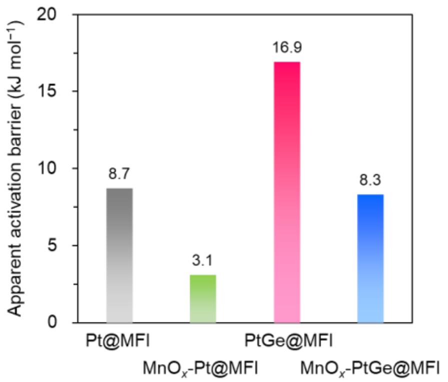

Figure S23. Summary of estimated apparent activation barriers for the H–D exchange.

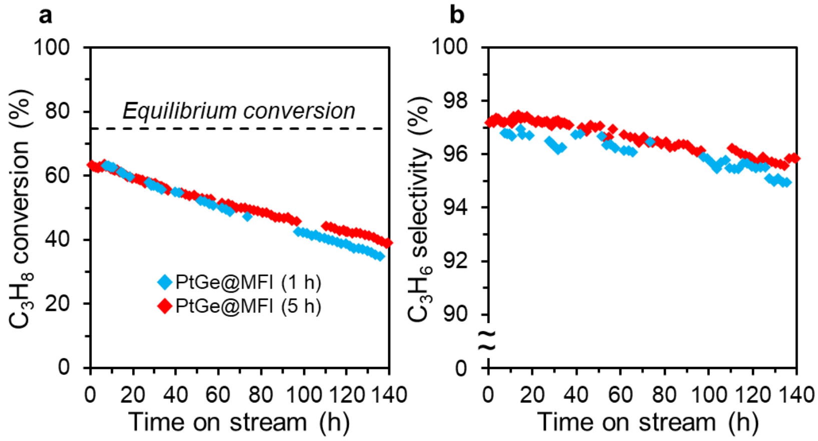

Figure S24. Catalytic performances of the PtGe@MFI catalysts with different reduction time (1 or 5 h at 700°C) in PDH at 630°C. Conditions: C_3H_8/He = 2.5/5.0, F = 7.5 mL min^{-1}, 30 mg of catalyst.

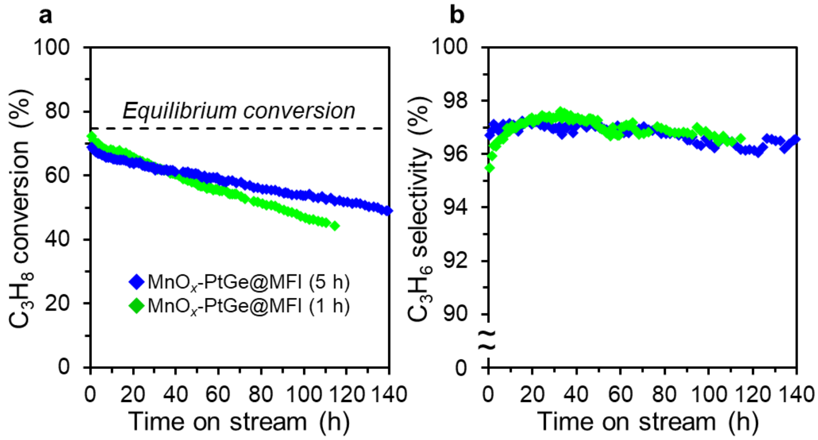

Figure S25. Catalytic performances of the MnOx-PtGe@MFI catalysts with different reduction time (1 or 5 h at 700°C) in PDH at 630°C. Conditions: C_3H_8/He = 2.5/5.0, F = 7.5 mL min^{-1}, 30 mg of catalyst.

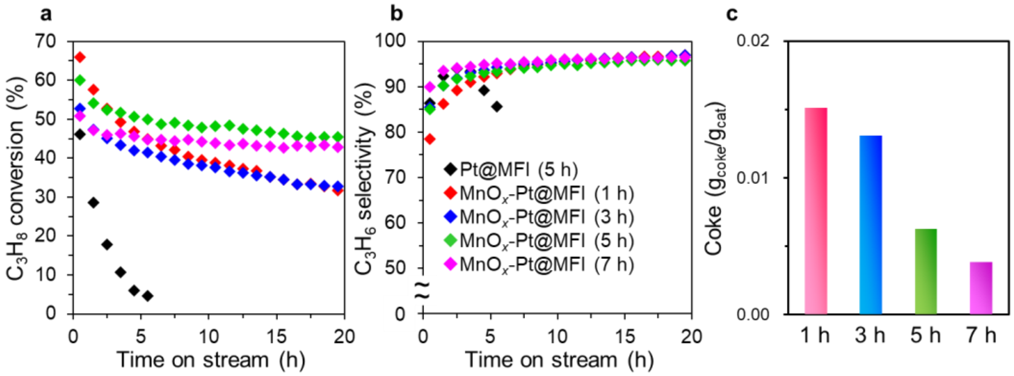

Figure S26. (a, b) Effect of reduction time for the as-prepared MnO_x-Pt@MFI catalysts. Conditions: 600°C, C_3H_8/He = 2.5/5.0, F = 7.5 mL min^{-1}, 50 mg of catalyst. (c) Coke amount estimated from the TPO profiles of the catalysts used in PDH at 600°C for 19.5 h.

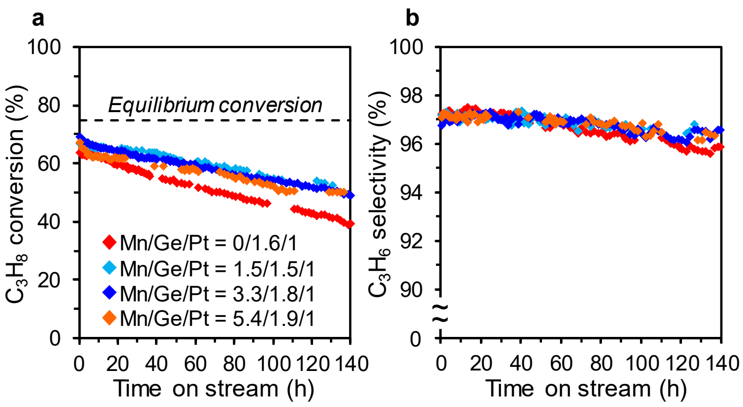

Figure S27. Catalytic performances of the MnO_x-PtGe@MFI catalysts with different Mn/Ge/Pt molar ratios in PDH at 630°C. Conditions: C_3H_8/He = 2.5/5.0, F = 7.5 mL min^{-1}, 30 mg of catalyst. The MnO_x-PtGeMn@MFI catalysts exhibited higher catalyst lifetimes compared with non Mn-contaning catalyst (PtGe@MFI) regardless of Mn amount, clearly indicating the positive role of Mn species in PDH.

Supplementary text 2. The effect of reduction time was investigated for PtGe@MFI and MnO_x-PtGe@MFI. The PtGe@MFI catalysts showed similar catalytic performance (Figure S24) for different reduction times (1 and 5 h at 700°C), whereas MnO_x-PtGe@MFI showed a clear difference in catalytic stability according to reduction time (Figure S25), with an improved catalytic stability at the longer reduction time. More distinct results were observed for MnO_x-Pt@MFI (Figure S26), which suggests that the oxidation state of MnO_x plays a significant role. One possible interpretation of these findings is that the formation of Pt–Ge alloy clusters and the partial reduction of MnO_x were complete within 1 h; however, the MnO_x species had to migrate to an optimized distribution, which took a longer time of more than 5 h at 700°C. We also studied the effect of Mn content (Figure S27) and observed that the Mn loading did not contribute to the PDH performance of MnO_x-PtGe@MFI. Therefore, the position of Mn species is also an important factor in greater catalytic performance.

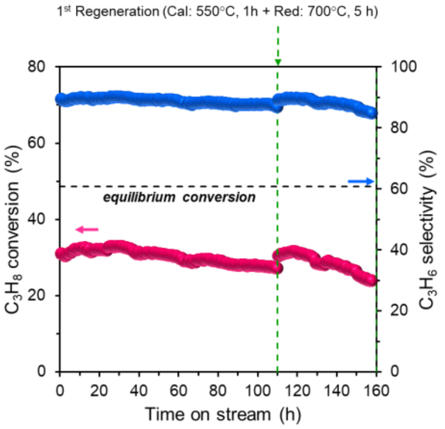

Figure S28. Catalytic performances of MnO_x-PtGe@MFI in PDH at 630°C. Conditions: C_3H_8 = 5.0 mL min^{-1}, 15 mg of catalyst. Regeneration process: 50%CO_2/He treatment at 550°C for 3 h; H_2 treatment at 700°C for 5 h.

Table S5. Summary of the catalytic performance of MnO_x-PtGe@MFI and other representative reported Pt-based catalysts in PDH in the absence of H_2.

entry	catalyst	temp. (°C)	conversion (%)[a]	gas composition	C_3H_6 sel. (%)[b]	specific activity (s^{-1})[c]	k_f (mol_{C3H6} g_{Pt}^{-1} h^{-1} bar^{-1})[d]	catalyst life τ (h)[e]	ref.
0-1	MnO_x-PtGe@MFI	630	31.1–27.2	C_3H_8 = 5	89.6–87.0	3.136	123.6	571	
0-2	MnO_x-PtGe@MFI	630	68.8–49.0	C_3H_8/He = 2.5/5	96.7–96.6	1.873	1377	167	
1	PtGe@MFI	630	63.5–39.2	C_3H_8/He = 2.5/5	97.2–95.8	1.622	619	140	This study
2	SnO_x-Pt@MFI	630	54.2–29.5	C_3H_8/He = 2.5/5	96.3–92.1	1.579	326	108	
3	(PtCoCu)(GeGaSn)/Ca–SiO_2: HEI	620	49.3 (11 h)–40.8	C_3H_8/He = 2.5/5	97.4–96.7	0.174	29	318	[9]
4	MnO_x-PtGe@MFI	600	59.9–46.0	C_3H_8/He = 2.5/5	98.8–99.1	0.999	722.7	1279	This study
5	SnO_x-Pt@MFI	600	49.3–45.2	C_3H_8/He = 2.5/5	99.0–99.1	0.886	195.7	229	
6	(PtCoCu)(GeGaSn)/Ca–SiO_2: HEI	600	40.3 (10 h)–31.2	C_3H_8/He = 2.5/5	99.3–98.7	0.145	20	628	[9]
7	PtGa–Ca–Pb/SiO_2	600	37.8 (8 h)-24.0	C_3H_8/He = 2.5/5	98.0–96.9	0.149	19	233	[23]
8	PtGa–Pb/SiO_2	600	31.6 (11 h)-14.5	C_3H_8/He = 2.5/5	97.9–94.3	0.125	13	149	[23]
9	1.5Ga0.1@S-1	600	45.9–41.5	C_3H_8/N_2 = 1/19	92.1	0.667	495	129	[24]
10	K-PtSn@MFI-600H_2-22h	600	38.7 (2 h)–31.9	C_3H_8/He = 24/76	>97	3.406	573	77	[2]
11	K-PtSn@MFI	600	20–17	C_3H_8/N_2 = 5/16.5	97	0.340	–	72	[1]
12	PtZn4@S-1-H	600	66.7–43.2	C_3H_8/N_2 = 1/3	90.8–95.5	0.370	834	60	[25]
13	Pt/Sn-ZSM-5	600	70–45	C_3H_8/N_2 = 1.5/5	99	0.472	–	23	[26]
14	0.3PtZn0.5@S-1	600	31.0–26.0	C_3H_8/N_2 = 11/19	97	1.044	99	16	[27]
15	Zn10Pt0.1/HZ	600	80–50	C_3H_8/N_2 = 5/95	56	0.130	476	14	[28]
16	$PtSnAl_{0.2}$/SBA-15	590	55.9–40.5	C_3H_8/Ar = 1/5	98.5	0.320	149	10	[29]
17	$PtSnAl_{0.1}$/SBA-15	590	55.1–38.8	C_3H_8/Ar = 1/5	97.8	0.313	139	9	[29]
18	0.7Pt0.7Zn/MZ	580	35-20	C_3H_8	98–95	0.782	–	626	[30]
19	PtGa–Ca–Pb/SiO_2	580	36.6-25.2	C_3H_8	98.0–97.0	0.087	–	465	[23]
20	PtLa/mz-deGa	580	42–417	C_3H_8	95	0.539	–	380	[31]
21	PtY/mz-deGa	580	42–5	C_3H_8	96	0.544	–	119	[31]
22	InPt/SSF	580	46.9–40.9	C_3H_8/Ar = 1/4	98	0.200	59	135	[32]
23	CePt/SSF	580	44.9–37.5	C_3H_8/Ar = 1/4	92	0.180	48	108	[32]
24	LaPt/SSF	580	44.4–35.2	C_3H_8/Ar = 1/4	92	0.178	46	86	[32]
25	FePt/SSF	580	56.8–44.0	C_3H_8/Ar = 1/4	90	0.223	143	64	[32]
26	PtSn/SiO_2	580	35.2–30.3	C_3H_8	>99	1.629	–	90	[6]
27	PtSn/SiO_2	580	34.5–27.6	C_3H_8	>9	2.434	–	62	[6]
28	Pt/0.8Sn-SBA-15	580	43.8–38.3	C_3H_8/Ar = 7/3	98.5	0.438	192	26	[33]
29	1Pt1Zn/MZ	580	30-27	C_3H_8	96–96	4.599	–	20	[30]
30	PtZn4@S-1-H	550	47.4–40.4	C_3H_8/N_2 = 1/3	93.2–99.2	0.270	337	759	[25]
31	PtZn4@S-1-H	550	40.0–21.8	C_3H_8/N_2 = 1/3	99.1–99.3	1.816	552	121	[25]
32	PtZn4@S-1-H	550	34.7–29.6	C_3H_8/N_2 = 1/3	99.1–98.6	3.151	633	41	[25]
33	PtZn4@S-1-H	550	21.2–11.8	C_3H_8/N_2 = 1/3	98.3–98.6	3.890	438	14	[25]
34	K-PtSn@MFI-600H_2-22h	550	20–17	C_3H_8	>97	7.040	822	351	[2]
35	$Ga^{\delta+}Pt^0$/SiO_2	550	40.7–38.5	C_3H_8/Ar = 1/4	63.5	0.016	5	217	[34]
36	$Ga^{\delta+}Pt^0$/SiO_2	550	36.5–26.9	C_3H_8/Ar = 1/4	90.9	0.408	99	45	[34]
37	$Ga^{\delta+}Pt^0$/SiO_2	550	31.9–18.2	C_3H_8/Ar = 1/4	99	0.847	170	27	[34]
38	0.1Pt-2Zn/Si-Beta	550	65–36	C_3H_8/He = 1/9	98	1.849	–	126	[35]
39	0.3Pt/0.5Sn-Si-Beta	550	27.5–25.2	C_3H_8/Ar = 1/19	99.1–99.9	0.152	82	101	[36]
40	$Pt^0Zn^{\delta+}$/SiO_2	550	35.3–26.6	C_3H_8/Ar = 1/4	97.6–96.3	1.147	261	73	[37]
41	$Pt^0Zn^{\delta+}$/SiO_2	550	30.2–16.1	C_3H_8/Ar = 1/4	98.1–97.0	0.422	76	37	[37]
42	Zn10Pt0.1/HZ	550	56.2–48.2	C_3H_8/N_2 = 5/95	78	0.127	–	62	[28]

[a] The first value was obtained at the beginning of the run, and the second at the end. [b] The C_3H_6 selectivity was obtained at the beginning of the run. [c] Defined as (mol of formed propylene per second) per mol of total Pt. [e] Although the k_f value was negative in some references, values are not listed in the table in such cases. This is mainly because the actual propane conversion was somehow higher than the estimated equilibrium conversion. [e] Mean catalyst life defined as $\tau = k_d^{-1}$.

Table S6. Summary of the catalytic performance of MnO_x-PtGe@MFI and other representative reported Pt-based catalysts in PDH in the absence of H_2.

entry	catalyst	Pt (wt%)	temp. (°C)	WHSV (h^{-1})[a]	k_d (h^{-1})[b]	operation time (h)[c]	ref
0-1	MnO_x-PtGe@MFI	0.43	630	39.4	0.002	571	This study
0-2	MnO_x-PtGe@MFI	0.43	630	9.8	0.006	140	
1	PtGe@MFI	0.46	630	9.8	0.007	140	
2	SnO_x-Pt@MFI	0.40	630	9.8	0.009	140	
3	(PtCoCu)(GeGaSn)/Ca–SiO_2: HEI	1	620	3.0	0.003	120	[9]
4	MnO_x-PtGe@MFI	0.43	600	5.9	0.001	720	This study
5	SnO_x-Pt@MFI	0.40	600	5.9	0.004	45	
6	(PtCoCu)(GeGaSn)/Ca–SiO_2: HEI	1	600	3.0	0.002	260	[9]
7	PtGa–Ca–Pb/SiO_2	3	600	9.8	0.004	160	[23]
8	PtGa–Pb/SiO_2	3	600	9.8	0.007	160	[23]
9	1.5Ga0.1@S-1	0.092	600	1.2	0.008	24	[24]
10	K-PtSn@MFI-600H2-22h	0.4	600	29.5	0.013	25	[2]
11	K-PtSn@MFI	0.42	600	1.8	0.014	67	[1]
12	PtZn4@S-1-H	0.72	600	3.6	0.017	58.3	[25]
13	Pt/Sn-ZSM-5	0.32	600	1.8	0.044	24	[26]
14	0.3PtZn0.5@S-1	0.23	600	6.5	0.061	5	[27]
15	Zn10Pt0.1/HZ	0.1	600	0.2	0.069	20	[28]
16	$PtSnAl_{0.2}$/SBA-15	0.5	590	2.4	0.104	6	[29]
17	$PtSnAl_{0.1}$/SBA-15	0.5	590	2.4	0.110	6	[29]
18	0.7Pt0.7Zn/MZ	0.7	580	13	0.002	480	[30]
19	PtGa–Ca–Pb/SiO_2	3	580	5.9	0.002	252	[23]
20	PtLa/mz-deGa	1	580	11.0	0.003	480	[31]
21	PtY/mz-deGa	1	580	11.0	0.008	312	[31]
22	InPt/SSF	1	580	3.5	0.007	33	[32]
23	CePt/SSF	1	580	3.5	0.009	33	[32]
24	LaPt/SSF	1	580	3.5	0.012	33	[32]
25	FePt/SSF	1	580	3.5	0.016	33	[32]
26	PtSn/SiO_2	0.5	580	19	0.011	20	[6]
27	PtSn/SiO_2	0.5	580	29	0.016	20	[6]
28	Pt/0.8Sn-SBA-15	1	580	8.3	0.038	6	[33]
29	1Pt1Zn/MZ	1	580	130	0.049	3	[30]
30	PtZn4@S-1-H	0.72	550	3.6	0.001	216.7	[25]
31	PtZn4@S-1-H	0.72	550	26.8	0.008	105.9	[25]
32	PtZn4@S-1-H	0.72	550	53.7	0.024	10.3	[25]
33	PtZn4@S-1-H	0.72	550	109.4	0.072	10.3	[25]
34	K-PtSn@MFI-600H2-22h	0.4	550	118.1	0.003	70	[2]

35	$Ga^{\delta+}Pt^{0}/SiO_2$	4.37	550	2.1	0.005	20	[34]
36	$Ga^{\delta+}Pt^{0}/SiO_2$	4.37	550	43.7	0.022	20	[34]
37	$Ga^{\delta+}Pt^{0}/SiO_2$	4.37	550	98.4	0.037	20	[34]
38	0.1Pt-2Zn/Si-Beta	1	550	2.4	0.008	150	[35]
39	0.3Pt/0.5Sn-Si-Beta	0.26	550	1.2	0.010	12	[36]
40	$Pt^{0}Zn^{\delta+}/SiO_2$	3.05	550	82.6	0.014	30	[37]
41	$Pt^{0}Zn^{\delta+}/SiO_2$	3.05	550	35.4	0.027	30	[37]
42	Zn10Pt0.1/HZ	0.1	550	0.2	0.016	20	[28]

[a] WHSV: weight hourly space velocity based on propane gas flow (h^{-1}). [b] The first-order deactivation model was used to estimate the catalyst stability.[4] [c] Operation time: total time tested for a single run.

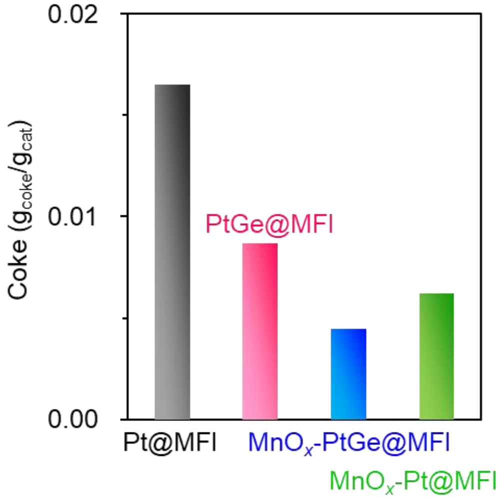

Table S7. Summary of the coke amount accumulated on the spent catalysts estimated by TPO.

Catalyst	Coke (g_{coke}/g_{cat})
Pt@MFI	0.016
PtGe@MFI	0.009
MnO*x*-PtGe@MFI	0.004
MnO*x*-Pt@MFI	0.006

Figure S29. Coke amount estimated from the TPO profiles of the catalysts used in PDH at 600°C for 19.5 h.

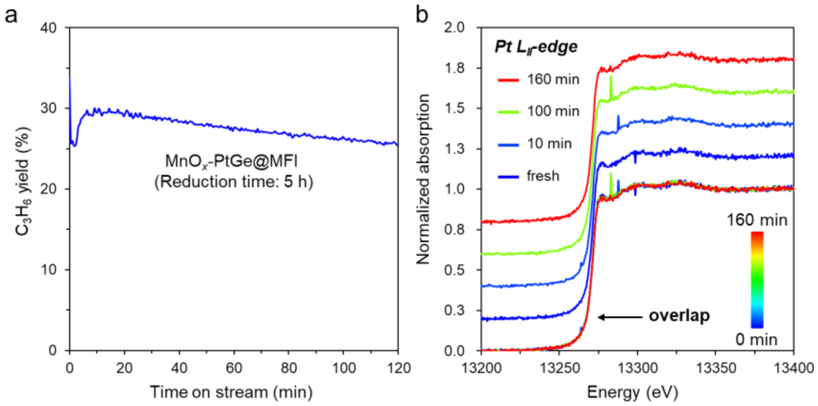

Figure S30. (a) C_3H_6 yield of the MnO$_x$-PtGe@MFI catalyst in PDH at 580°C. Conditions: C_3H_8/Ar = 5/10, F = 15 mL min^{-1}, 30 mg of catalyst. (b) Pt L_{II}-edge XANES spectra of the MnO$_x$-PtGe@MFI catalyst recorded at 600°C under at continuous flow of propane. Conditions: C_3H_8/He = 3/47, F = 50 mL min^{-1}, 60 mg of catalyst.

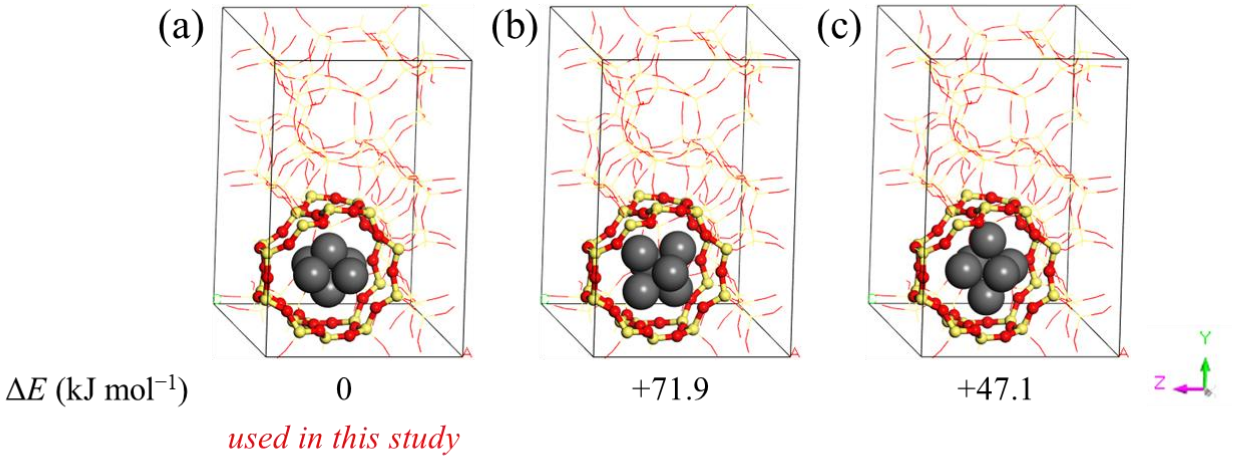

Figure S31. Difference in the electronic energies of various configurations of the MFI-encapsulated Pt_6 clusters. The energy of the lowest one (used as a model structure in this study) was set to zero.

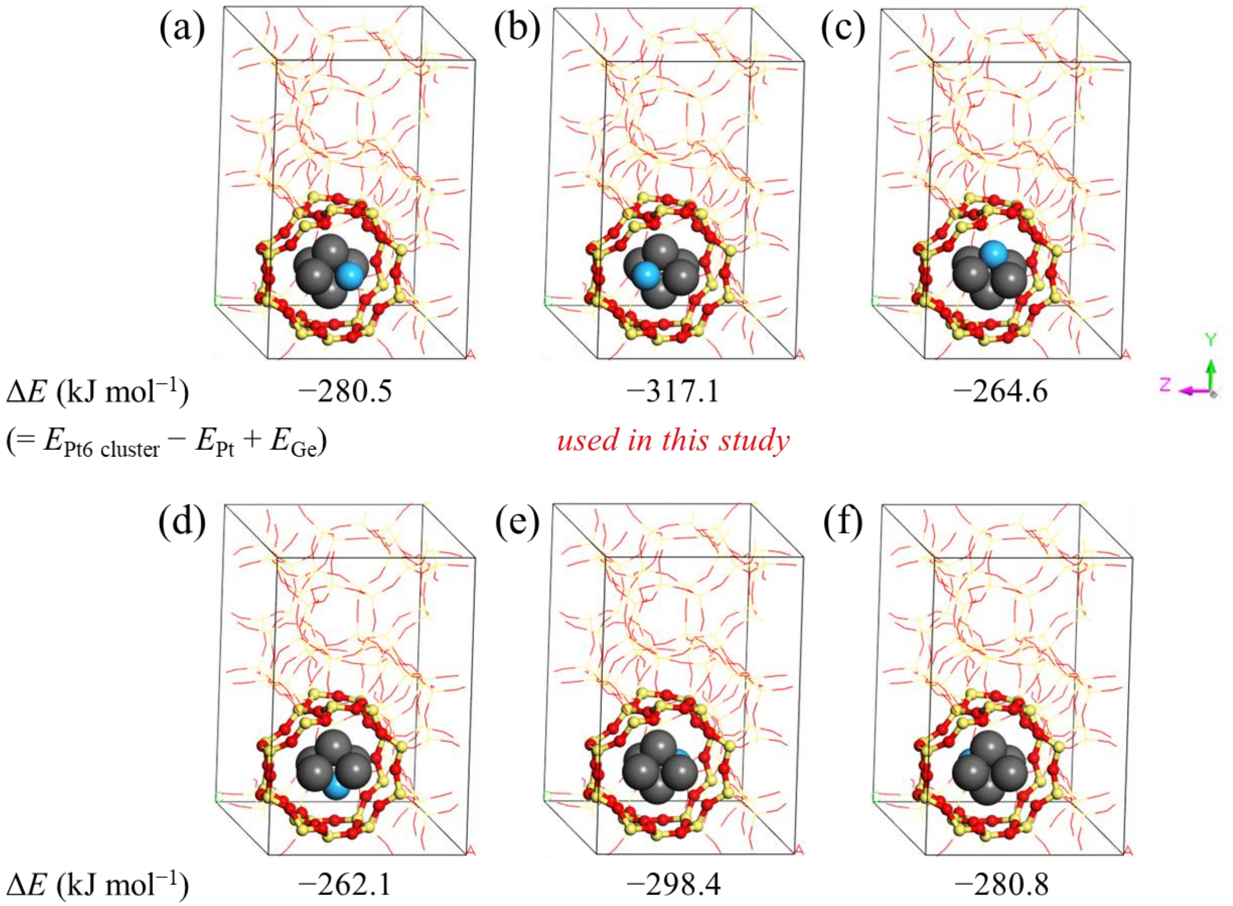

Figure S32. Difference in the electronic energies of various configurations of the MFI-encapsulated Pt_5Ge_1 clusters. The structure, which has lowest energy was used as a model structure in this study. All Pt_5Ge_1@MFI models were made by replacing one Pt atom in the Pt_6@MFI model structure with one Ge atom. The substitution of Pt with Ge was largely exoenergetic ($\Delta E = -317$ kJmol^{-1}), which suggested that the alloying (which is an entropic process) was thermodynamically favorable.

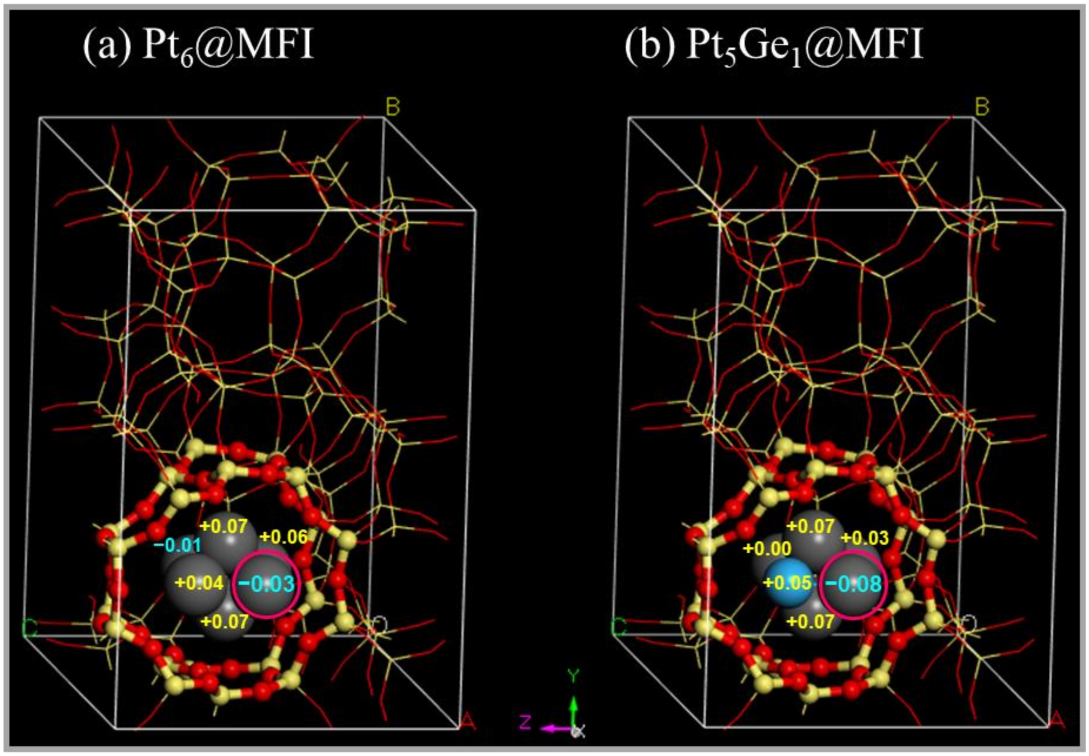

Figure S33. Hirshfeld charges of (a) Pt_6@MFI and (b) Pt5Ge_1@MFI.

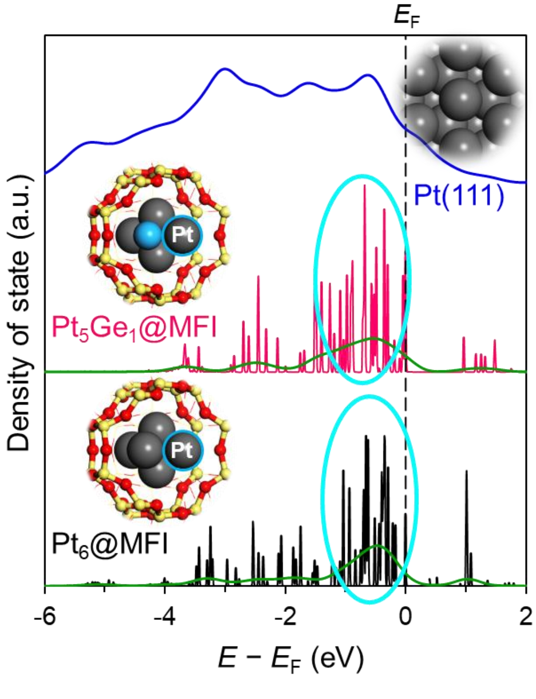

Figure S34. Density of states (DOS) projected on *d* orbitals of the Pt sites (designated by light blue circles) in Pt_6@MFI and Pt_5Ge_1@MFI. For comparison, *d* structure of Pt sites on the Pt(111) surface is shown.

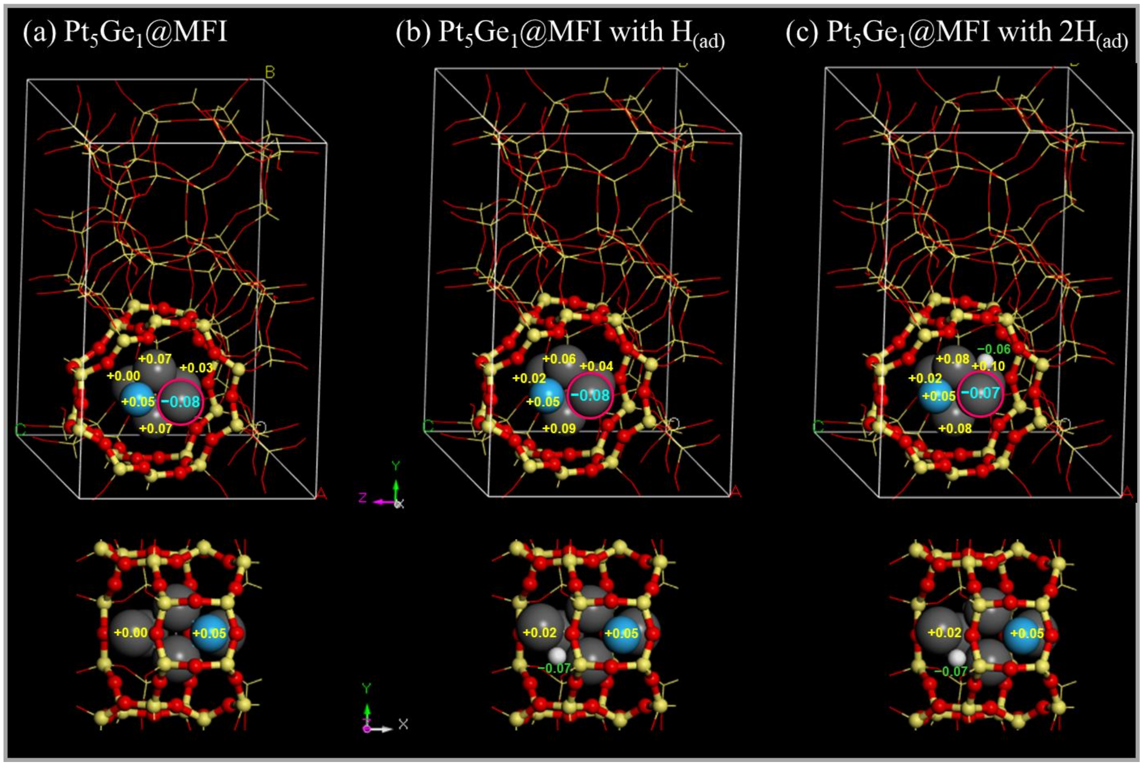

Figure S35. Density of states (DOS) projected on d orbitals of the Pt sites (designated by pink circles) in Pt_5Ge_1@MFI. (a) without, (b) with one, and (c) with two co-adsorbed hydrogen atoms on Pt_5Ge_1 cluster.

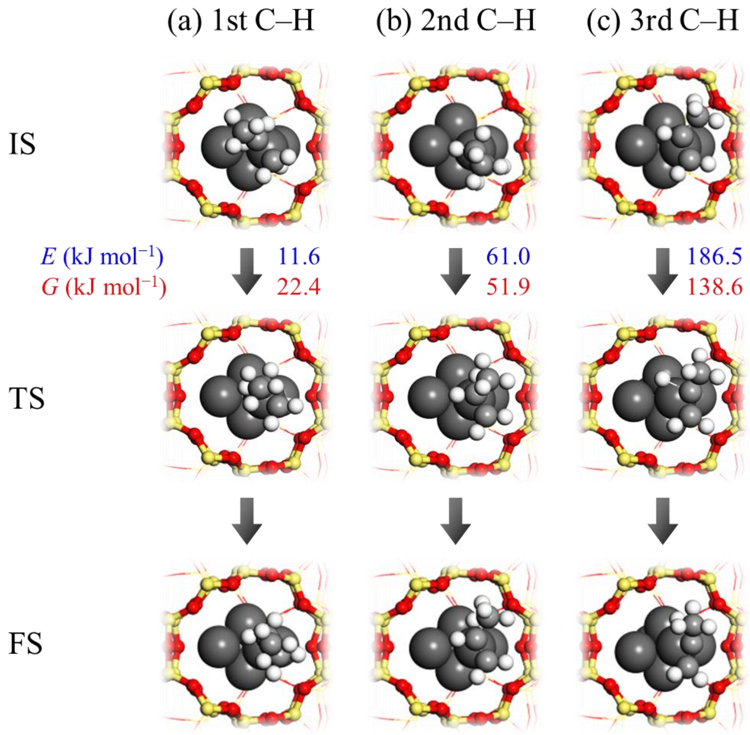

Figure S36. Structures of initial (IS), transition (TS), and final states (FS) of (a) 1st, (b) 2nd, and (c) 3rd C–H scissions in PDH on the Pt_6 cluster.

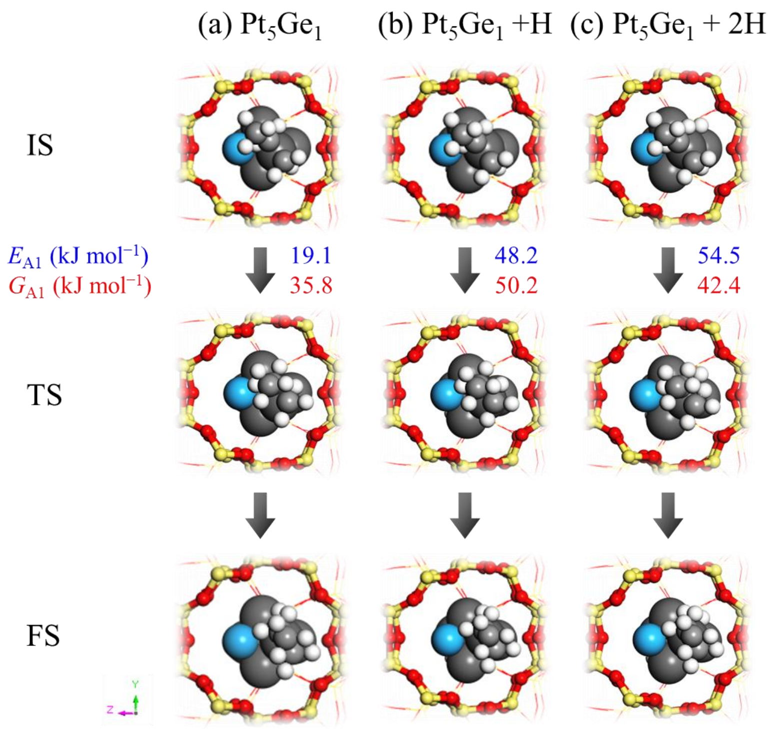

Figure S37. Structures of initial (IS), transition (TS), and final states (FS) of 1st C–H scission in PDH on the Pt_5Ge_1 clusters (a) without, with (b) one and (c) two co-adsorbed hydrogen atoms.

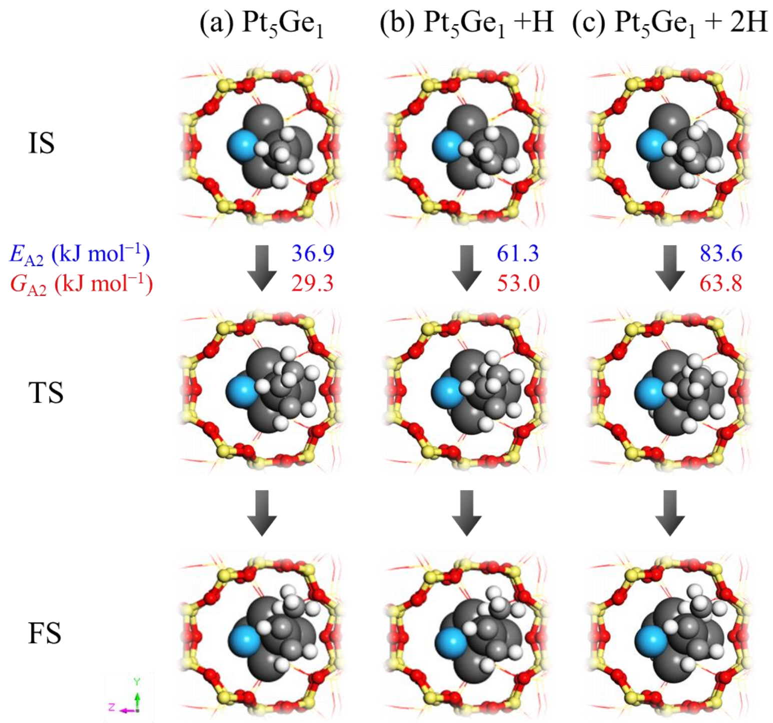

Figure S38. Structures of initial (IS), transition (TS), and final states (FS) of 2nd C–H scission in PDH on the Pt_5Ge_1 clusters (a) without, with (b) one and (c) two co-adsorbed hydrogen atoms.

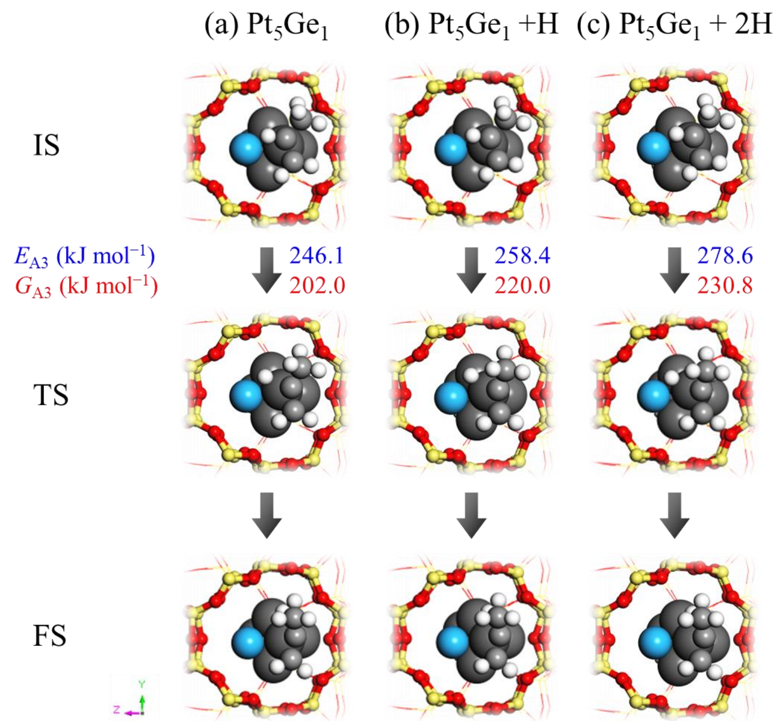

Figure S39. Structures of initial (IS), transition (TS), and final states (FS) of 3rd C–H scission in PDH on the Pt_5Ge_1 clusters (a) without, with (b) one and (c) two co-adsorbed hydrogen atoms.

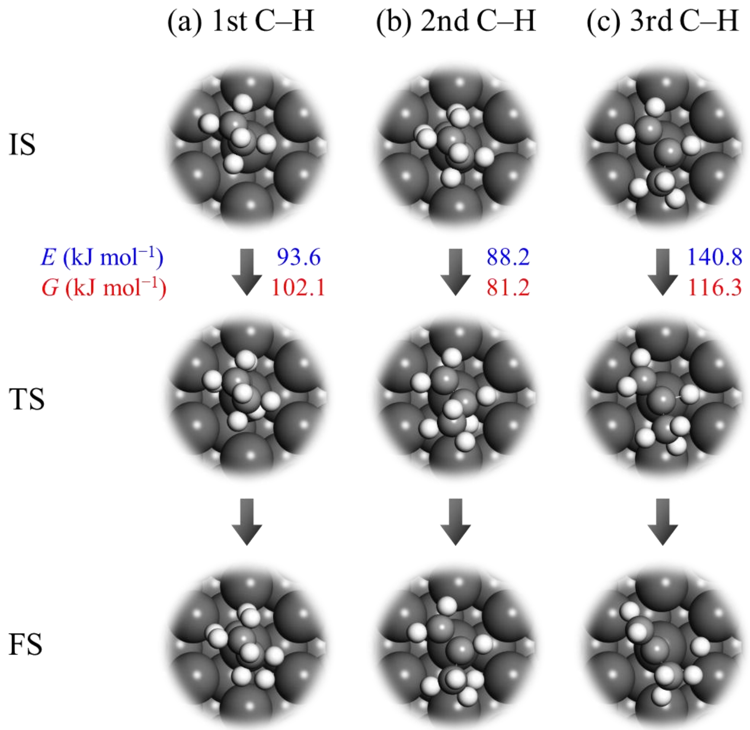

Figure S40. Structures of initial (IS), transition (TS), and final states (FS) of (a) 1st, (b) 2nd, and (c) 3rd C–H scissions in PDH on the Pt(111) site.

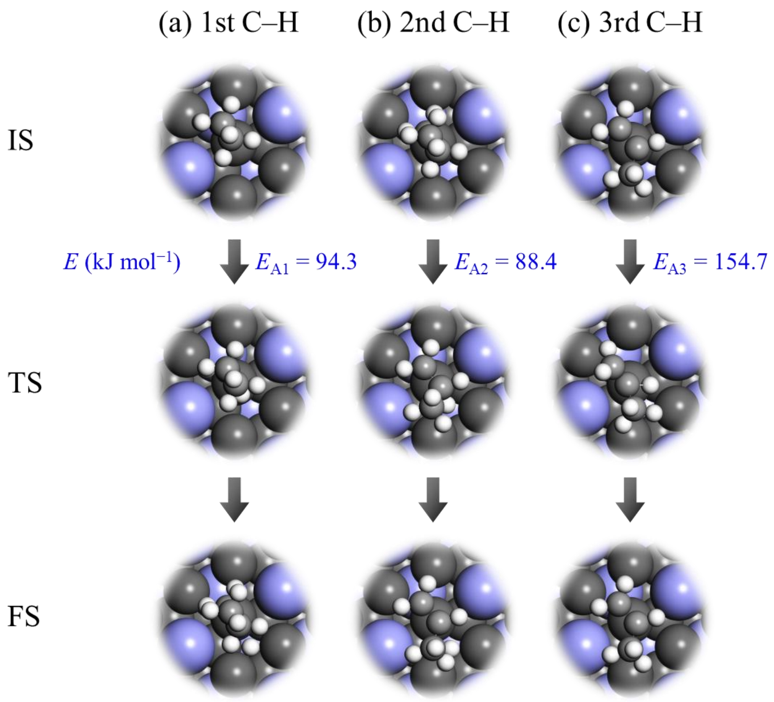

Figure S41. Structures of initial (IS), transition (TS), and final states (FS) of (a) 1st, (b) 2nd, and (c) 3rd C–H scissions in PDH on the $Pt_3Sn(111)$ site.

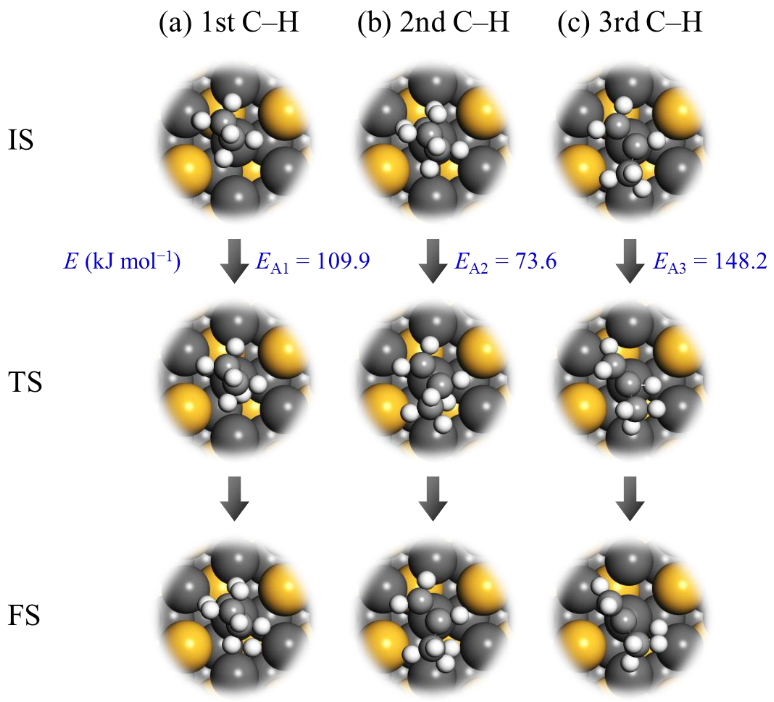

Figure S42. Structures of initial (IS), transition (TS), and final states (FS) of (a) 1st, (b) 2nd, and (c) 3rd C–H scissions in PDH on the $Pt_3In(111)$ site.

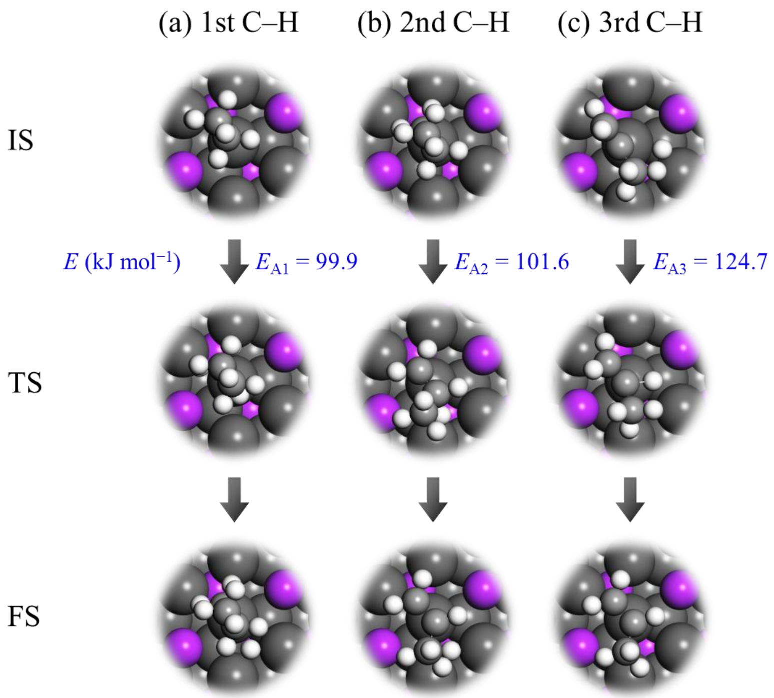

Figure S43. Structures of initial (IS), transition (TS), and final states (FS) of (a) 1st, (b) 2nd, and (c) 3rd C–H scissions in PDH on the $Pt_3Mn(111)$ site.

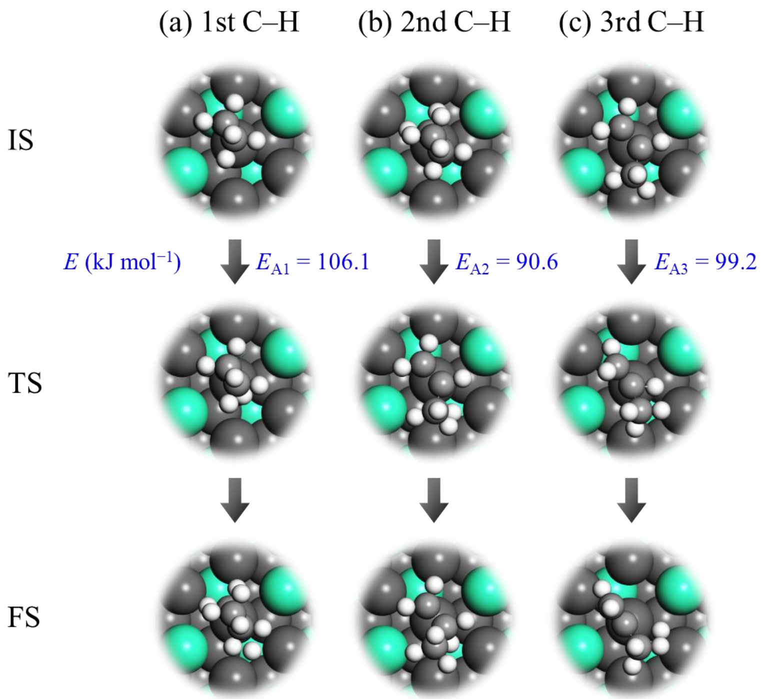

Figure S44. Structures of initial (IS), transition (TS), and final states (FS) of (a) 1st, (b) 2nd, and (c) 3rd C–H scissions in PDH on the $Pt_3Y(111)$ site.

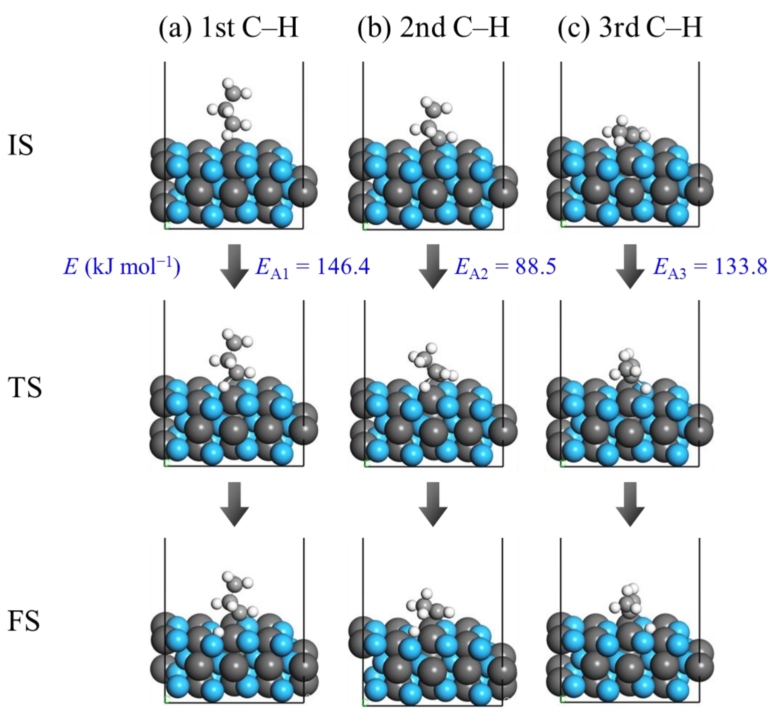

Figure S45. Structures of initial (IS), transition (TS), and final states (FS) of (a) 1st, (b) 2nd, and (c) 3rd C–H scissions in PDH on the PtGe(020) site.

Table S8. Summary of DFT calculation for PDH on various metallic surfaces.

Models	Energy barrier (kJ mol^{-1})			Adsorption energy (kJ mol^{-1})		ΔE[b]
	E_{A1}	E_{A2}	E_{A3}	$E_{ad\text{-}propane}$	$E_{ad\text{-}propylene}$[a]	
Slab						
Pt(111)	93.6	88.2	140.8	−20.1	−79.9	60.9
$Pt_3Sn(111)$	94.3	88.4	154.7	−8.3	−65.7	89.1
$Pt_3In(111)$	109.9	73.6	148.2	−22.8	−71.5	76.7
$Pt_3Mn(111)$	99.9	101.6	124.7	−70.5	−90.0	34.7
$Pt_3Y(111)$	106.1	90.6	99.2	−17.3	−54.0	45.2
PtGe(020)	146.4	88.5	133.8	−13.8	−31.1	102.7
Cluster						
Pt_6 cluster	11.6	61.0	186.5	−27.2	−161.4	25.2
Pt_5Ge_1 cluster	19.1	36.9	246.1	−6.8	−141.6	104.4
Pt_5Ge_1 cluster with $H_{(ad)}$	48.2	61.3	258.4	−19.6	−164.7	93.6
Pt_5Ge_1 cluster with $2H_{(ad)}$	54.5	83.6	278.6	−3.8	−183.0	95.6

[a]$E_{ad} = -E_d$. [b]$\Delta E = E_{A3} + E_{ad} = E_{A3} - E_d$.

Table S9. Summary of DFT calculation for PDH on various metallic surfaces.

Models	Free energy barrier (kJ mol^{-1})			G_{ad}	ΔG[a]
	G_{A1}	G_{A2}	G_{A3}		
Cluster					
Pt_6 cluster	22.4	51.9	138.6	−0.1	138.7
Pt_5Ge_1 cluster	35.8	29.3	202.0	17.3	219.3
Pt_5Ge_1 cluster with $H_{(ad)}$	50.2	53.0	220.0	−16.2	203.7
Pt_5Ge_1 cluster with $2H_{(ad)}$	42.4	63.8	230.8	−64.9	165.9

[a] $\Delta G = G_{A3} + G_{ad} = G_{A3} - G_{d}$

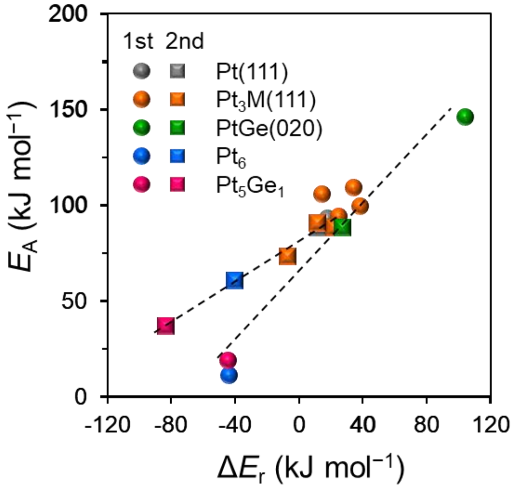

Figure S46. Relationships between ΔE_r and E_A.

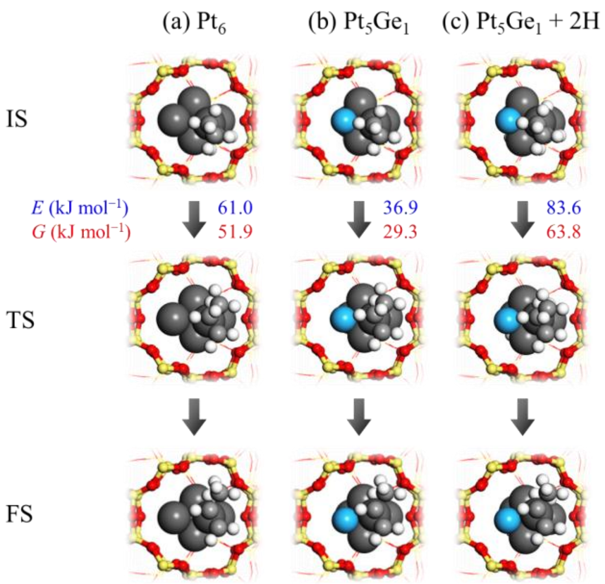

Figure S47. Structures of initial (IS), transition (TS), and final states (FS) of 2nd C–H scission in PDH on the (a) Pt_6, (b) Pt_5Ge_1, and (c) Pt_5Ge_1 with two co-adsorbed hydrogen atoms. The corresponding structures are also shown in Figures S36 and S38.

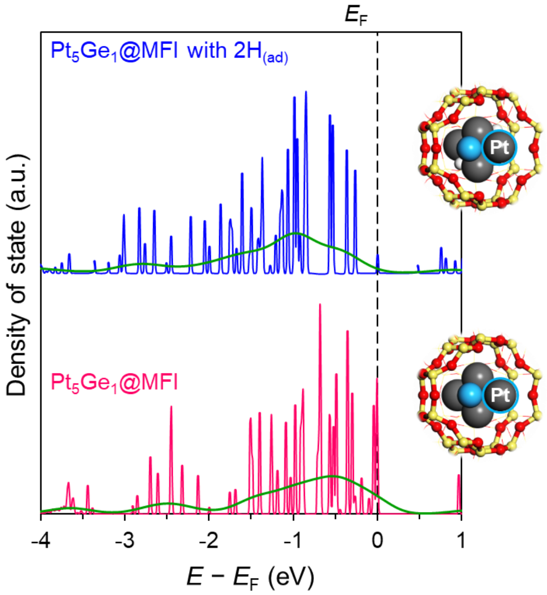

Figure S48. Density of states (DOS) projected on *d* orbitals of the Pt sites in Pt_5Ge_1@MFI with and without two co-adsorbed hydrogen atoms. Green lines indicate the broadened curves for visibility.

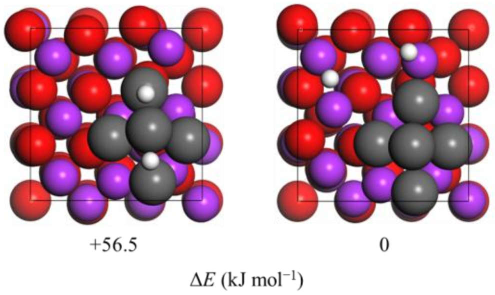

Figure S49. The adsorption energies of two hydrogen atoms on Pt/MnO.

Supplementary text 3.

Why the PtGe clusters shows high activity?

It can be explained by the Brønsted−Evans−Polanyi (BEP) relationship. In our calculations, the changes in E_A consistently followed the BEP relationship in all considered structures (Figure S46). This means that the reaction energy (ΔE_r) of C–H scission, *i.e.*, the energy difference between the initial and final states, determines the energy barrier. The *d* electron levels of a Pt atom in Pt_6 and Pt_5Ge_1 were obviously upshifted relative to those of Pt(111) (Figure S34), resulting in greater adsorptivity. In this context, the final states of the 1st/2nd C–H scissions (C_3H_7+H_2/C_3H_6+H_2) were highly stabilized, thereby lowering the E_A values. Furthermore, when comparing the Pt_6 and Pt_5Ge_1 clusters, Pt_5Ge_1 exhibited lower E_{A2} value (rate-determining step) than Pt_6. Upon the incorporation of Ge, the C_3H_7 adsorption (initial state of 2nd C–H scission) was destabilized, leading to the decrease in E_{A2} (Figure 3c). Whereas no significant difference was observed in the adsorption conformation of C_3H_7 (Figure S47), the d electron levels for Pt_5Ge_1 were shifted downward from the Fermi level. Therefore, the weakening of C_3H_7 adsorption can be

attributed to the electronic effect by Ge. Thus, "downsizing" and "alloying with Ge" synergistically contributed to the lowering of the E_A value of RDS, which enables the outstanding activity.

The prevention of hydrogen poisoning on PtGe clusters by MnO$_x$

As revealed by experiments, excess hydrogen not only reduces the number of available active sites on Pt–Ge clusters but also increases E_{app} values (Figure 2e). To confirm this phenomenon using DFT calculations, we investigated the influence of co-adsorbed hydrogen (H(a)) on the Pt_5Ge_1 cluster. Even in the presence of H(a), the TS structure changed little (C–H distance at TS: 1.58 Å [bare] → 1.56 Å [2H(a)]), which suggests that there was no geometric effect of H(a). Conversely, hydrogen adsorption downshifted the *d* electron levels (Figure S48) likely owing to the stabilization of *d* electrons by Pt–H bond formation. Therefore, the addition of two hydrogen on Pt_5Ge_1 made the initial state (C_3H_7(a)) of the 2nd C–H scission highly stable than the final state, increasing the E_{A2} value from 36.9 to 83.6 kJ mol^{-1} (Figures 4c–d and Figures S37–39). Therefore, reducing the concentration of H(a) on the clusters is essential to achieving the potentially high catalytic activity of Pt_5Ge_1, which can be achieved by placing MnO_x in close proximity to the clusters. A simplified Pt/MnO model was employed for qualitative understanding, which revealed that adsorption of two hydrogen atoms onto MnO was 56.5 kJ mol^{-1} more stable than onto Pt (Figure S49). This strongly suggests that MnO_x act as a hydrogen trap to decrease its coverage on the PtGe clusters.

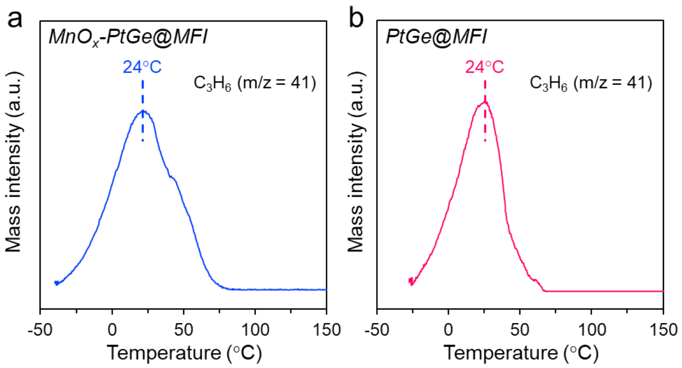

Figure S50. C_3H_6-TPD for the (a) MnO_x-PtGe@MFI and (b) PtGe@MFI catalysts (adsorption temperature: −35°C).

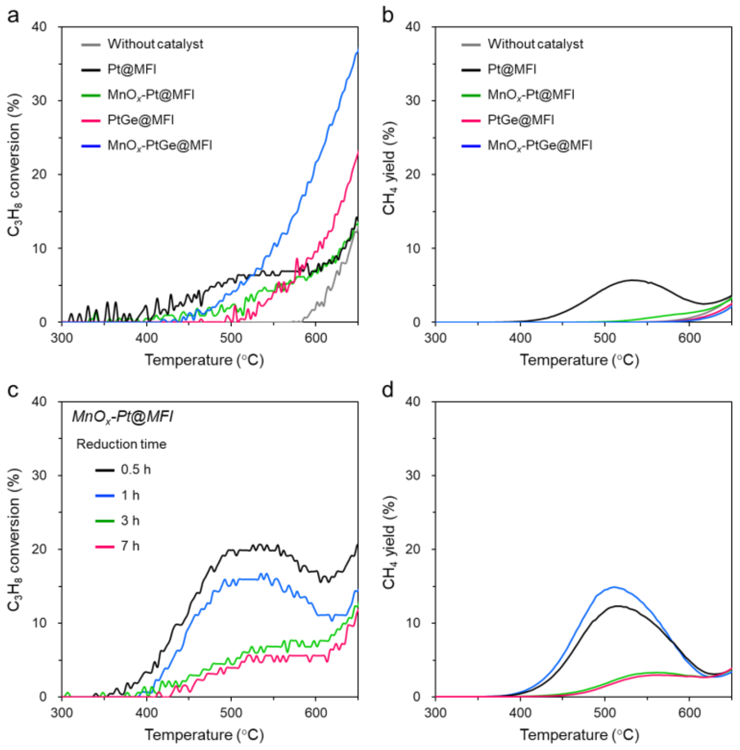

Figure S51. (a) C_3H_8 conversion and (b) CH_4 yield for the Pt@MFI, MnO_x-Pt@MFI, PtGe@MFI, and MnO_x-PtGe@MFI catalysts. (c) C_3H_8 conversion and (d) CH_4 yield for the MnO_x-Pt@MFI catalysts with the different reduction time. Conditions: $C_3H_8/H_2/Ar = 1/1/19$, $F = 21$ mL min^{-1}, 10 mg of catalyst.

Supplementary References

[1] L. Liu, M. Lopez-Haro, C. W. Lopes, C. Li, P. Concepcion, L. Simonelli, J. J. Calvino, A. Corma, *Nat. Mater.* **2019**, *18*, 866–873.

[2] L. Liu, M. Lopez-Haro, C. W. Lopes, S. Rojas-Buzo, P. Concepcion, R. Manzorro, L. Simonelli, A. Sattler, P. Serna, J. J. Calvino, A. Corma, *Nat. Catal.* **2020**, *3*, 628–638.

[3] L. Liu, M. Lopez-Haro, C. W. Lopes, D. M. Meira, P. Concepcion, J. J. Calvino, A. Corma, *J. Catal.* **2020**, *391*, 11–24.

[4] J. J. H. B. Sattler, J. Ruiz-Martinez, E. Santillan-Jimenez, B. M. Weckhuysen, *Chem. Rev.* **2014**, *114*, 10613–10653.

[5] L. Qi, M. Babucci, Y. Zhang, A. Lund, L. Liu, J. Li, Y. Chen, A. S. Hoffman, S. R. Bare, Y. Han, B. C. Gates, A. T. Bell, *J. Am. Chem. Soc.* **2021**, *143*, 21364–21378.

[6] A. H. Motagamwala, R. Almallahi, J. Wortman, V. O. Igenegbai, S. Linic, *Science* **2021**, *373*, 217–222.

[7] B. Ravel, M. Newville, *J. Synchrotron Radiat.* **2005**, *12*, 537–541.

[8] A. Ankudinov, B. Ravel, *Phys. Rev. B - Condens. Matter Mater. Phys.* **1998**, *58*, 7565–7576.

[9] Y. Nakaya, E. Hayashida, H. Asakura, S. Takakusagi, S. Yasumura, K. Shimizu, S. Furukawa, *J. Am. Chem. Soc.* **2022**, *144*, 15944–15953.

[10] H. Funke, M. Chukalina, "Wavelet Transform for EXAFS," can be found under https://www.esrf.fr/UsersAndScience/Experiments/CRG/BM20/Software/Wavelets, **n.d.**

[11] H. Funke, A. C. Scheinost, M. Chukalina, *Phys. Rev. B* **2005**, *71*, 94110.

[12] H. Funke, M. Chukalina, A. C. Scheinost, *J. Synchrotron Radiat.* **2007**, *14*, 426–432.

[13] M. Filez, E. A. Redekop, H. Poelman, V. V Galvita, R. K. Ramachandran, J. Dendooven, C. Detavernier, G. B. Marin, *Chem. Mater.* **2014**, *26*, 5936–5949.

[14] M. D. Segall, P. J. D. Lindan, M. J. Probert, C. J. Pickard, P. J. Hasnip, S. J. Clark, M. C. Payne, *J. Phys. Condens. Matter* **2002**, *14*, 2717–2744.

[15] B. Hammer, L. B. Hansen, J. K. Nørskov, *Phys. Rev. B - Condens. Matter Mater. Phys.* **1999**, *59*, 7413–7421.

[16] A. Tkatchenko, M. Scheffler, *Phys. Rev. Lett.* **2009**, *102*, 6–9.

[17] K. Hu, M. Wu, S. Hinokuma, T. Ohto, M. Wakisaka, J. I. Fujita, Y. Ito, *J. Mater. Chem. A* **2019**, *7*, 2156–2164.

[18] T. A. Halgren, W. N. Lipscomb, *Chem. Phys. Lett.* **1977**, *49*, 225–232.

[19] N. Govind, M. Petersen, G. Fitzgerald, D. King-Smith, J. Andzelm, *Comput. Mater. Sci.* **2003**, *28*, 250–258.

[20] J. De Graaf, A. J. Van Dillen, K. P. De Jong, D. C. Koningsberger, *J. Catal.* **2001**, *203*, 307–321.

[21] Y. Nakaya, J. Hirayama, S. Yamazoe, K. Shimizu, S. Furukawa, *Nat. Commun.* **2020**, *11*, 2838.

[22] L. Liu, D. M. Meira, R. Arenal, P. Concepcion, A. V. Puga, A. Corma, *ACS Catal.* **2019**, *9*, 10626–10639.

[23] Y. Nakaya, F. Xing, H. Ham, K. Shimizu, S. Furukawa, *Angew. Chem. Int. Ed.* **2021**, *60*, 19715–19719.

[24] Y. Wang, Y. Suo, X. Lv, Z. Wang, Z. Y. Yuan, *J. Colloid Interface Sci.* **2021**, *593*, 304–314.

[25] Q. Sun, N. Wang, Q. Fan, L. Zeng, A. Mayoral, S. Miao, R. Yang, Z. Jiang, W. Zhou, J. Zhang, T. Zhang, J. Xu, P. Zhang, J. Cheng, D. C. Yang, R. Jia, L. Li, Q. Zhang, Y. Wang, O. Terasaki, J. Yu, *Angew. Chem. Int. Ed.* **2020**, *59*, 19450–19459.

[26] J. Zhu, R. Osuga, R. Ishikawa, N. Shibata, Y. Ikuhara, J. N. Kondo, M. Ogura, J. Yu, T. Wakihara, Z. Liu, T. Okubo, *Angew. Chem. Int. Ed.* **2020**, *59*, 19669–19674.

[27] Y. Wang, Z. P. Hu, X. Lv, L. Chen, Z. Y. Yuan, *J. Catal.* **2020**, *385*, 61–69.

[28] C. Chen, M. Sun, Z. Hu, J. Ren, S. Zhang, Z. Y. Yuan, *Catal. Sci. Technol.* **2019**, *9*, 1979–1988.

[29] X. Fan, J. Li, Z. Zhao, Y. Wei, J. Liu, A. Duan, G. Jiang, *Catal. Sci. Technol.* **2015**, *5*, 339–350.

[30] S. W. Han, H. Park, J. Han, J. Kim, J. Lee, C. Jo, R. Ryoo, *ACS Catal.* **2021**, *11*, 9233–9241.

[31] R. Ryoo, J. Kim, C. Jo, S. W. Han, J. C. Kim, H. Park, J. Han, H. S. Shin, J. W. Shin, *Nature* **2020**, *585*, 221–224.

[32] Y. Qiu, X. Li, Y. Zhang, C. Xie, S. Zhou, R. Wang, S. Z. Luo, F. Jing, W. Chu, *Ind. Eng. Chem. Res.* **2019**, 10804–10818.

[33] B. Li, Z. Xu, W. Chu, S. Luo, F. Jing, *Cuihua Xuebao/Chinese J. Catal.* **2017**, *38*, 726–735.

[34] K. Searles, K. W. Chan, J. A. Mendes Burak, D. Zemlyanov, O. Safonova, C. Copéret, *J. Am. Chem. Soc.* **2018**, *140*, 11674–11679.

[35] L. Xie, Y. Chai, L. Sun, W. Dai, G. Wu, N. Guan, L. Li, *J. Energy Chem.* **2021**, *57*, 92–98.

[36] Y. Wang, Z. P. Hu, W. Tian, L. Gao, Z. Wang, Z. Y. Yuan, *Catal. Sci. Technol.* **2019**, *9*, 6993–7002.

[37] L. Rochlitz, K. Searles, J. Alfke, D. Zemlyanov, O. V. Safonova, C. Copéret, *Chem. Sci.* **2020**, *11*, 1549–1555.
